# Implementing patient-centred outcome measures in palliative care clinical practice. An updated systematic review of facilitators and barriers

**DOI:** 10.1186/s12904-026-01997-2

**Published:** 2026-02-12

**Authors:** Bárbara Antunes, Stephen Barclay, Isla Kuhn, Kathy Eagar, Claudia Bausewein, Fliss Murtagh, Simon Etkind, Ben Bowers, Sarah Dixon, Roberta Lovick, Richard Harding, Irene Higginson, Farhad Shokraneh

**Affiliations:** 1 Palliative and End of Life Care Group in Cambridge (PELiCam), Department of Public Health and Primary Care, Primary Care Unit, The University of Cambridge, Forvie Site, Robinson Way, Cambridge, CB2 0SR UK; 2 Medical Library, School of Clinical Medicine, The University of Cambridge, Forvie Site, Robinson Way, Cambridge, CB2 0SR UK; 3 Queensland University of Technology, 2 George St, Brisbane City , QLD 4000 Australia; 4 Department of Palliative Medicine, LMU Munich, Munich University Hospital, Campus Großhadern | Marchioninistr, 15, Munich, 81377 Germany; 5 Wolfson Palliative Care Research Centre, University of Hull, Allam Medical Building, Hull, HU6 7RX UK; 6 Department of Palliative Care and Cicely Saunders Institute, King’s College London, Bessemer Rd, London, SE5 9RS UK; 7 Department of Evidence Synthesis, Systematic Review Consultants LTD, 57 Woodstock Rd, Oxford, OX2 6HJ UK

**Keywords:** Patient-centred outcome measurement, Patient-reported outcome measurement, Palliative care, Systematic reviews, Implementation, Barriers, Facilitators, Complex interventions

## Abstract

**Background:**

Patient-centred outcome measures (PCOMs), when well implemented, are powerful tools facilitating patient, family and clinical communication to better respond to patient needs. Their routine use in palliative care practice still faces challenges.

**Objective:**

To update a systematic review of PCOMs implementation, reviewing and synthesising new evidence on facilitators, barriers, lessons learned, measures used, models of implementation, costs, implementation outcomes, and consequences in clinical practice.

**Methods:**

We searched eight information sources supplemented by hand-searching and citations of the original review and studies identified by the expert advisory committee. This prospectively registered review included studies using a PCOM during clinical care of adult patients with advanced disease in all settings and extracted data on: PCOMs used, models of implementation, facilitators, barriers, lessons learned, costs, and implementation outcomes. We employed narrative synthesis and tabulated findings, following all PRISMA reporting guidelines.

**Results:**

We included 114 studies. A major new facilitator was the integration of electronic/digital PCOMs into Information Technology systems. Main barriers remain largely unchanged and relate to healthcare professionals’ beliefs. Implementation was highlighted as a complex intervention, needing planning, assessment and fine tuning throughout. Sixty-two included studies mentioned at least one implementation outcome. Eighteen models, frameworks and theories were identified in 25 included studies. No studies reported on costs of implementation.

**Conclusion:**

This work reveals the complexity of implementing PCOMs in palliative care practice. The main clinical and research implications of our findings highlight the central importance of staff engagement and training staff in PCOM tools, communication strategies, and cultural competence.

**Supplementary Information:**

The online version contains supplementary material available at 10.1186/s12904-026-01997-2.

## Key Statements

### What is already known about the topic

- The importance of using patient-centred outcome measures (PCOMs) to understand the effect and effectiveness of health interventions has been established: they are an essential component of evidence-based clinical practice.
- Data collected at individual patient-level can be used immediately by healthcare professionals to act on any identified distressing symptoms or palliative needs.
- Despite the development of PCOMs in the past three decades, their routine use in clinical practice remains challenging in most countries.

### What this paper adds

- Having a coordinator onsite and training clinicians are key to the initial implementation of PCOMs in clinical practice.
- Embedding PCOMs at point of care is critical to their ongoing implementation in practice.
- The integration of electronic/digital PCOMs into Information Technology systems is a new development that is still emerging
- Evidence is scarce on the models of PCOMs implementation used in clinical practice and their associated financial costs.

### Implication for practice, theory or policy

- Successful implementation requires embedding outcome measures at the point of care and tailoring the implementation process to local circumstances and contexts.
- Constant feedback of patient-level outcomes to clinicians is vital.
- Implementing PCOMs in palliative care clinical practice is a complex intervention and guidelines and statements on complex interventions should be followed.

## Background

The importance of patient-centred outcome measures (PCOMs) in palliative care clinical practice is well established: they include patient-reported outcome measures (PROMs), which capture patients’ own perspectives on their health and wellbeing, and together they allow for clinical care to respond to real-time need, and longitudinal comprehensive measurement of patient and family outcomes across physical, psychological, existential, emotional, and practical domains [1–5].

When used properly, PCOMs become powerful communication tools, keeping all involved in patient care at the same level of knowledge regarding patient and family outcomes [6–8]. Additionally, PCOMs data, collected at patient level, can be aggregated for audit, research, quality improvement, and benchmarking. Ultimately those data can be used by policy makers to improve patient and family care [9–11].

Regardless of the recognition and importance PCOMs have in patient and family care, their implementation in clinical practice remains challenging [12, 13]. Time pressures in busy clinical settings, limited clinician training and confidence in using and interpreting PCOM data, and concerns about increasing workload are some of the issues. Additionally, organisational and system-level factors—such as inadequate leadership support and poor integration into existing clinical processes—continue to impede sustainable implementation [1].

We have updated and expanded a 2013 systematic review on implementation of PCOMs in palliative care [1]. The original review objectives were to (a) identify barriers and facilitators to the systematic implementation of PROMs in palliative care clinical practice, (b) identify needs and other comments of clinical teams regarding the routine use of PROMs and (c) identify lessons learned on the process of implementation of PROMs in clinical practice. Twenty-six studies were included within 31 articles. We updated the original review objectives and recommendations and added four more objectives, with review questions as follows:

1. What are the PCOMs implemented in palliative care clinical practice?
2. What are the facilitators to PCOMs implementation in palliative care clinical practice?
3. What are the barriers to PCOMs implementation in palliative care clinical practice?
4. What are the lessons learned on implementing PCOMs in PC clinical practice?
5. What are the implementation models used in PCOMs implementation in palliative care clinical practice?
6. What implementation outcomes were measured and how, when implementing PCOMs in palliative care clinical practice?
7. What are the financial costs of implementing PCOMs in palliative care clinical practice?
8. Are there new recommendations since the previous literature review to inform the implementation process in palliative care clinical practice for all stakeholders?

## Methods

We conducted a systematic literature review and narrative synthesis. PRISMA reporting guidelines were followed, including PRISMA, PRISMA-Abstracts [14] and PRISMA-Search [15]. The review protocol has been published elsewhere in open access [16]; we briefly describe the methods below.

On 14 November 2022, we searched MEDLINE, Embase, Emcare, CINAHL, PsycINFO, BNI, Web of Science Core Collections (SSCI, SCI, ESCI), and Scopus with no date, language, publication type, or publication status limitations [17], supplemented by a list of studies recommended by the expert advisory committee and hand-searching references of included studies. Appendix 1 provides the full search strategy for MEDLINE. We included primary empirical studies using PCOMs in clinical care for adult patients with advanced disease in all palliative care settings. We included studies using a PCOM during clinical care of adult patients with advanced disease in all settings and extracted data on: PCOMs used, models of implementation, facilitators, barriers, lessons learned, costs, and implementation outcomes. Narrative reviews, editorials, commentaries, and case reports were excluded.

The search results were imported into EndNote and de-duplicated. Following the removal of duplicates, approximately 20,000 records were screened. The de-duplicated results were then exported from EndNote and imported into Rayyan (Ouzzani et al., 2016) for screening. Two reviewers (B.A., F.S.) independently screened 20% of the records using the blinding mode. When inter-reviewer agreement exceeded 90%, the remaining records were screened by a single reviewer. Any discrepancies between reviewers were resolved through discussion. All full-text reports were independently assessed by two reviewers, and any unresolved disagreements were adjudicated by a senior topic expert, who made the final decision regarding study eligibility.

We used Gough’s Weight of Evidence Framework [18] to appraise each included study’s internal validity, appropriateness, and contribution to addressing the review questions. The framework applies to quantitative, qualitative, and mixed-methods research at the study level. The weight of evidence refers to the preponderance of evidence used to inform decision-making. It involves judgements on general methodological quality (Weight of Evidence A), the appropriateness of the research design for the review question (Weight of Evidence B), and the relevance or focus of the evidence to the review (Weight of Evidence C). Each of these dimensions was rated as low, medium, or high. These three judgements were then combined to generate an overall assessment (Weight of Evidence D), also categorised as low, medium, or high, reflecting the strength and relevance of each study’s contribution to the synthesis. Studies were weighted independently by two reviewers (B.B., S.E.), with a third reviewer (S.B.) consulted when necessary to reach consensus.

The narrative synthesis was undertaken in the same way, following the Guidance on the Conduct of Narrative Synthesis in Systematic Reviews [19]. Finally, we mapped the updated recommendations, and reiterated those from our previous review, on the updated Medical Research Council (MRC) Framework for Developing and Evaluating Complex Interventions [20]. The Framework has four phases, each with six core elements.

We updated the original 2022 search by a scoping search using our main keywords on 9 February 2025 to identify any new relevant studies.

## Results

We included 114 studies (117 reports) [2, 7, 8, 13, 21–133] which are shown in Table 1.

**Table 1:** Characteristics and quality assessment of included studies

Study Name	Research Date	Country	Design	Setting	Participants (Patient, Carer, HCP)	Age (Mean (SD))	Sex/Gender; *N* (%)	Size	Conditions	WoE
Appleyard et al. 2021	2016–2017	UK	Single-Arm Interventional Study (Mixed-Method)	Uro-Oncology Clinics (Outpatient Department) of 1 Teaching & 2 District General Hospitals; Home (Optional)	Patients (Computer-Familiar)	M (R): 74 (58–89	M	40	Advanced Prostate Cancer on Palliative Systemic Treatment	HHH-H
Bausewein et al. 2011a	2009–2010	Africa, EU, Malaysia, Taiwan, Australia, Brazil, US, Canada, Japan, Thailand	Survey (Mixed Methods)	NA	HCP	46 (9)	F: 420; M: 243	663	NA	HHH-H
Bausewein et al. 2016	2011–2013	EU	Concensus (EAPC White/Position Paper)	NA	HCP	NA	NA	28	NA	LLL-L
Bausewein et al. 2018	2014	Germany	Case Study	1 university palliative care unit (10-bedded)	Patients	NA	F/M	574	Advanced cancer; advanced lung and heart disease; neurological disease	LMM-M
Beddard-Huber et al. 2015	2013	Canada	Single-Arm Interventional Study; Survey	1 Tertiary Palliative Care Unit (15-bed palliative care unit at Vancouver General Hospital); Acute	Patients, HCP: Nurses, Physicians	Median: 68.6 (Patients)	F/M	92 Patients, 23 Nurses, 7 Physicians	Cancer (84%); Cardiac disease (4%); End-stage liver disease (3%); Other chronic diseases (8%); Brain injury (1%)	LLL-L
Bennett et al. 2012	NA	UK, US	Survey	Oncology (Hospice, Hospital, Home)	NA	NA	F/M	NA	Oncology (Treatment and survivorship, Adults and adolescents receiving chemotherapy and palliative care, Postsurgery for radical prostatectomy, In hospice)	LMH-M
Bookbinder et al. 1996	1992–1994	US	Pre-Post Interventional Study; Focus Groups; Chart Reviews	Major Urban Cancer Center: 12 inpatient units	Patients; HCP: Nurse	NA	NA	398 Patients; Nurses (NA)	Cancer Pain (acute and chronic)	MMM-M
Bourbonnais et al. 2004	NA	Canada	Retrospective Chart Reivew; Focus group	12 settings	Patients; HCP: Nurses	NA	NA	55 Patients; 25 Nurses	Cancer Pain	HHH-H
Bouvette et al. 2002	2000	Canada	Exploratory study; Focus groups; Chart audits	12: Community agencies (5); Tertiary Hospital (2); Community Hospital (2); Hospices (2); ORPCU (1)	HCP: Nurses	NA	NA	180	Lung cancer; Breast cancer; Prostate cancer; Stomach and peritoneal cancer; Lower back pain; Oesophagus, larynx, parotid cancer; Brain cancer; CHF, MI, angina, COPD, emphysema; Liver or pancreas cancer; Cervix cancer; Diabetes; Lymphoma; Myeloma	HHH-H
Bradshaw et al. 2021b	2019	UK	Qualitative Study (Exploratory (Interpretive Paradigm)	11 services delivering specialist palliative care in Yorkshire, England (inpatient, outpatient/day therapy, home-based/community)	HCP: Nurse (29); Doctor/consultant (16); AHP (8); Healthcare assistant (4); Chief executive (2); IT (2); Other (2)	25–55+	F (59); M (4)	63	NA	HHH-H
Bush et al. 2018	NA	NA	Systematic Review	NA	NA	NA	NA	30 primary studies	NA	LHM-M
Campbell et al. 2022	NA	US	Prospective Observational Study; Interviews	2 outpatient gynecologic oncology clinics at a large urban academic medical center	Patients	60.6 (10.7)	F	30	Gynecologic cancer receiving chemotherapy: Ovarian/fallopian; Primary peritoneal, uterine, endometrial, or cervical; Other/unknown	MLL-L
Carli Buttenschoen et al. 2014	2010	Canada	Survey	Palliative Community Consult Team (1 20-bed Tertiary Palliative Care Unit, 3 Hospice Palliative Care Units, 1 Community Consultation Team, and 3 Acute Care Hospital Palliative Consultation Teams) and University Hospital Chronic Pain Clinic; Inpatient (hospital, hospice); outpatient clinics; home consultations	HCP: Staff physician: 11; Fellows, residents, students: 11; Nurses: 51; Other (dieticians, occupational therapists, pharmacists, physiotherapists, respiratory therapists, and spiritual care): 10	NA	NA	193	Palliative care; Chronic pain	MMM-M
Coast et al. 2018	2012–2014	UK	Qualitative Study; Interviews; Constant Comparative Analysis; Feasibility	1 UK Adult Hospice (patients recruited via community service, day hospice and in-patient unit)	33 hospice patients, 22 close persons and 17; HCP: 8 doctors, 7 nurses and 2 AHP	50+ (13 aged 50–69, 10 aged 70–79, 10 aged ≥ 80 years)	F: 12	72	Life-threatening illness and approaching EoL: Cancer-related diagnoses; motor neurone disease	MML-M
Collins et al. 2015	2010–2014	NA	Systematic Review	Palliative Care	NA	NA	NA	43 studies (POS *n* = 35, STAS *n* = 8)	Cancer, HIV/AIDS, Dementia, Parkinson’s Disease, Chronic Kidney Disease, Chronic Heart Failure, COPD, Renal Transplant Patients, End-Stage Renal Disease, Parkinson Syndromes (Idiopathic Parkinson’s Disease, Progressive Supranuclear Palsy, and Multiple System Atrophy)	HHM-H
Collins et al. 2015	NA	NA	Systematic Review	Palliative Cancer Care	NA	NA	NA	13 primary studies	NA	HHH-H
Currow et al. 2014	2008	Australia	Prospective Database Analysis (Symptom Control Performance Data)	30 Services (Metropolitan; Regional/rural): Inpatient, Ambulatory/community, Both	Patients	NA	F: NA (46)	19,747	Cancer: 85%	HHH-H
Daveson et al. 2012	NA	Europe and Africa	Survey (Web-Based)	NA	HCP: Palliative care professionals working in clinical care, audit and research	Doctors: 46.9 (8.74); Nurses: 45.1 (8.42)	Doctors: F: 92 (47); Nurses: F: 87 (84)	196 Doctors; 104 Nurses	NA	MMM-M
Diffin et al. 2018	2013–2014	UK	Pre-Post Single-Arm Interventional Study; Survey	36 UK palliative care services: Day services, Community clinical nurse specialist teams, day hospices, social work teams, and an outpatient clinic	HCP: Clinical Nurse Specialist (CNS) (73); Registered Nurse (other than CNS) (37); Social Worker (7); Medical Practitioner (3); Other (34)	NA	NA	462	NA	HHH-H
Diplock et al. 2019	2011–2013	Canada	Prospective Cohort Study	1 Ambulatory Clinics in a Regional Cancer Center	Patients	Control: 58.33 (16.06); Case 61.19 (12.99)	M: Control − 94 (58.75); Case − 28 (25.93); F: Control − 66 (41.25); Case − 80 (74.07)	268: Control (*n* = 160) and Case (*n* = 108)	Breast, Gastro-intestinal, Genitourinary, Gynecology, Hematology oncology, Head and neck, Lung, Melanoma	MMM-M
Dobrina et al. 2018	2012–2014	Italy	Mixed-Method; Single-Arm Interventional Study; Action Research	1 Italian hospice (19 beds)	Patients; HCP	NA	NA	16 patients; 2 Physicians (1 Chief), 1 Chief Nurse, 10 RNs, 10 NAs, 1 Psychologist (Patient and Family Support), 1 Psychologist (Staff Support)	advanced cancer	MML-M
Donaldson et al. 2004	NA	NA	Literature Review; Interviews	Oncology Practice; hospice, home-care, and long-term care settings	NA	NA	NA	NA	Cancer	HHH-H
Downing et al. 2012	2010	Africa	Survey (Web-Based)	NA	HCP: 69 (Physician: 27; Nurse: 29; Other: 13); Researcher: 27 (Pure Researcher: 9; Researcher-Clinician: 18)	47 (9)	F: 55; M: 33	301	NA	MHH-H
Dunckley et al. 2005	NA	UK	Pre-Post Interventional Study; Action Research; Interviews; Case Studies; Staff Diaries	1 specialist hospice inpatient unit and a nursing home providing care for people with neurological illness	HCP	NA	NA	28 permanent nursing home staff and to 23 clinical hospice staff	NA	HMM-M
Eijsink et al. 2023	NA	NA	Scoping Review	NA	NA	NA	NA	20 studies	Bariatric surgery; Pain interference in carpal tunnel release; Ischemic stroke; Minor stroke and transient ischemic attack; Total knee and hip replacement; Aortic valve disease; Hernia care; Hepatatis C; Orthopedic surgery; Coronary artery disease; Breast cancer; Breast cancer; Inflammatory arthritis; Turner syndrome; IBD; Lynch syndrome; Burn injury; Cancer care; Hemodialysis; Advanced non-small-cell lung cancer	HMM-M
Ellis-Smith et al. 2018	2014–2016	UK	Mixed-Method; Feasibility	3 residential care homes registered to provide care for people aged 65 and over (26–33 beds)	6 family members and 20 professionals care home staff (*n* = 15), GPs (*n* = 3) and DNs (*n* = 2)	87.2 (8.3)	F: 24 (75); M: 8 (25)	32 Residents (Dementia with comorbidities)	Dementia	HHH-H
Etkind et al. 2015	1985–2013	NA	Systematic Review	Outpatient oncology (Mainly); Community oncology; Home hospice patients; Hospice inpatient unit; Long-term care units; Outpatient oncology; Palliative home care	NA	NA	NA	16 papers corresponding to 13 studies	NA	HHH-H
Evans et al. 2020	2017–2018	Canada	Mixed-Methods; Pre-Post Study; Survey; Interviews; Chart Audit; Pilot; Longitudinal Study	8 in-facility hemodialysis programs	Patients, Carers, HCP (48 providers and staff: nephrologists (*n* = 5), nurse practitioners (*n* = 3), nurse managers (*n* = 5), registered nurses and registered practical nurses (*n* = 7), pharmacists (*n* = 3), dietitians (*n* = 9), social workers (*n* = 4), clerical staff (*n* = 3), and project leads (*n* = 9))	M: 68 (1459 patients)	NA	9 patients/caregivers and 48 HCPs; 1207 charts (1459 patients)	Hemodialysis	MHH-H
Fabian et al. 2021	NA	NA	Systematic Review	Palliative Radiotherapy	NA	NA	NA	4 studies (primary objective); 3 studies (secondary objective)	Head and Neck Cancer Patients	HMM-M
Fetz et al. 2018	2011–2015	Germany	Observational Cohort study; Retrospective Analysis of Longitudinal Data	Specialised Palliative Care Unit of a University Medical Centre	HCP: Nurses	M (R; SD): 67 (17–114; 14)	F: 420; M: 400	820	Cancer; Non-cancer; Previous cancer	MMM-M
Friedman et al. 2022	2013–2019	US	Prospective Single-Arm Interventional Study	Hematology-oncology clinics in Veteran Administration (VA) medical centers in the Southeast	Patients	M (R): 67 (21–97)	M: 8,106 (89); F: 952 (11)	9,058	Ambulatory patients: No retrievable cancer diagnosis; Prostate cancer; Lung cancer; Leukemias and lymphomas; Colorectal cancer; Head and neck cancer; Multiple myeloma; Hepatocellular carcinoma; Renal cell carcinoma; Breast cancer; Pancreatic cancer; Other cancers	MML-M
Friis et al. 2021	2014–2018	Denmark	Prospective Single-Arm Cohort	1 oncology outpatient	Patients	Median (IQR): 70 (64–74)	F: 35 (37.2); M: 59 (62.8)	94	Advanced Lung Cancer: SCLC, NSCLC	HML-M
Gabbard et al. 2021	2017–2018	US	Feasibility	1 outpatient dialysis unit affiliated with a large academic tertiary medical center	Patients	69.4 (6.6)	F: 14 (63.6); M: 8 (36.4)	22	End-Stage Renal on Hemodialysis Disease; Charlson Comorbidity Index was 8.45 (2.28)	HHH-H
Garcia et al. 2019	2015–2017	US	Prospective Database Analysis	1 comprehensive cancer center	Patients	57.15 (13.39)	F: 2398 (68.11)	6825 (3521 Responders)	Oncology outpatients; Hematologic malignancies (lymphoma and leukemia), Breast, Gynecologic malignancies, Gastrointestinal malignancies, Sarcoma (soft tissue and bone), Lung and other thoracic malignancies, Other or unspecified malignancies, Prostate, Skin, Other genitourinary, Central nervous system, Head and neck, Thyroid and other endocrine glands, Missing	HMM-M
Goyal et al. 2020	2013–2014	US	Prospective Pre-Post Study	1 radiotherapy outpatient setting	Patients	64.3 (12.2)	F: 106 (42); M: 149 (58)	255	Tumor Primary disease site: Breast, Head and neck, Lung, Prostate, GI, Lymphoma, Sarcoma, CNS, Gynecology, Other	HMM-M
Graf et al. 2022	2015–2016	Germany	Prospective Trial	2 centers in major university hospitals	Patients	Adjuvant Therapy (*n* = 76, AGE: 49.39 (10.28)); Metastatic Situation (*n* = 30, AGE: 53.93 (13.94))	F	106	Adjuvant or advanced breast cancer; Adjuvant Therapy (*n* = 76, AGE: 49.39 (10.28)); Metastatic Situation (*n* = 30, AGE: 53.93 (13.94))	MHL-M
Greenhalgh et al. 2017	NA	NA	Realist Synthesis	NA	NA	NA	NA	NA	NA	HMM-M
Gressel et al. 2019	2016–2017	US	Prospective Cross-Sectional Study; Pilot	1 Gynecologic oncology outpatient clinic	Patients	65 (12)	NA	336	Uterine cancer, Ovarian/fallopian/PPC, Cervical/vaginal/vulvar cancer	HMM-M
Guo et al. 2018	2017	Asia	Workshop Report	NA	HCP: doctors, nurses, AHP; Managers, policy makers, and academics	NA	NA	350	NA	LLM-L
Hall et al. 2020	2018	UK	Qualitative Study; Longitudinal Study	1 NHS Trust	HCP	NA	NA	12 hospital practitioners, 1 hospital administrator and 4 community practitioners (= 17 (then 15): 4 Nurses Community; 8 Nurses; 3 Consultants, Matron, Project Officer Hospital)	NA	HMM-M
Harding et al. 2007	2005	South Africa	Workshop Report	NA	HCP	NA	NA	32: doctor (*n* = 11), service manager/developer (*n* = 6), nurse (*n* = 6), social/support worker (*n* = 5), trainer/educator (*n* = 2), technical advisor (*n* = 1), and medical student (*n* = 1)	NA	MHH-H
Harding et al. 2011	2009	EU	Survey (Online)	Palliative and Advanced Disease Care	HCP: Physicians, nurses, other allied professionals, and academics	45.8 (8.93)	F: 187 (63.6)	311	NA	HMM-M
Hardy et al. 1999	NA	UK	Pre-Post Single-Arm Interventional Study	1 palliative care unit	Patients	Median (R): 66 (36–83)	NA	52	Advanced disease	MMM-M
Hawley et al. 2011	NA	Canada	Pre-Post Controlled Interventional Study; Retrospective Chart Review	3 outpatient oncology pain and symptom management/palliative care clinics, 4 palliative care units (general hospitals), 4 residential hospices, 1 control palliative care unit	HCP: 15 members of clinical staff: 9 registered nurses, 4 licensed practical nurses, 1 physician, and 1 other.	NA	NA	180 charts	Constipation in patients receiving palliative care: Mostly cancer patients (Breast, lung, gastrointestinal, and prostate primaries)	MMM-M
Hill et al. 2002	NA	New Zealand	Qualitative Study; Theme of Revelation (De Santis and Ugarriza)	Hospice setting	HCP; Patients	NA	NA	NA	NA	MLL-L
Hogberg et al. 2019	2016–2017	Sweden	Qualitative Study: Interpretive Descriptive Design; Interviews (Telephone)	3 Specialized palliative home-care	Patients	M (R): 72 (46–85)	F: 3; M: 7	10	Incurable cancer; Advanced cancer (9), stroke (1)	HHH-H
Howell et al. 2020	2014–2016	Canada	Observational Pre-Post Controlled Population Cohort Study	8 disease site clinics at 3 regional cancer centers (RCCs)	Patients	M, Median (IQR): 62.69, 64 (19)	F: 35,122; M: 35,732	70,854	Brain cancer, Breast cancer, Lung cancer, Gynecological cancer, Colorectal cancer, Prostate cancer, Other cancer	HHH-H
Hughes et al. 2003*	NA	UK	Qualitative Study; Interviews (Telephone); Exploratory	NA	HCP	NA	NA	26	NA	HHM-H
Hughes et al. 2004*	NA	UK	Qualitative Study; Exploratory Scoping Exercise	10 settings: 4 oncology wards, 4 nursing homes, 1 general medical ward, 1 specialist hospital palliative care team	HCP	NA	NA	NA	NA	MML-M
Hui et al. 2017a	NA	US	Narrative Review	NA	NA	NA	NA	NA	NA	MLL-L
Hui et al. 2017b	2015	US	Pre-Post Interventional Study	1 Community Cancer Center (General Medical Oncology Outpatient Clinic)	Patients	Average (R) 55 (19–87)	F: 265 (57)	465	Breast, Gastrointestinal, Genitourinary, Head and neck, Hematological, Other, Respiratory Cancers	HMM-M
Ihler et al. 2019	2017	Norway	Qualitative Study; Interviews (Interpretive)	2 Oncology Wards in a Hospital; Inpatients	HCP: 6 RN (5 Oncology Nurse)	Average (R): 37.2 (28–49)	F	6	Cancer	MMM-M
Jordhoy et al. 2007	2005	Norway	Systematic Review	NA	NA	NA	NA	40 references	NA	MMM-M
Kamal et al. 2016	2014–2015	US	Prospective Cross-Sectional Study	Palliative care (Diverse Clinical Settings) 5 organizations within the Palliative Care Research Cooperative Group: 4 academic sites and 1 community-based site; across 6 common clinical settings of palliative care: hospital general floor, hospital intensive care unit (ICU), emergency department, outpatient, long-term care, and home; (general floor, ICU, and emergency department) as acute care; outpatient palliative care clinics, home-based palliative care consultations, and long-term care facility palliative care visits were aggregated into nonacute care	Patients	NA	F: 1228 (55); M: 961 (43); Left blank: 53 (2)	2242	Cancer, Neurologic, Cardiovascular, Pulmonary, Infectious, Other diagnosis, Gastrointestinal, Renal, Unknown	HHH-H
Kane et al. 2017*	2014–2015	Ireland	Mixed Method; Pre-Post Study; Qualitative Study; Interviews	CHF disease management clinics in 2 national tertiary referral centres in Dublin, Ireland; urban teaching hospital sites	Patients (18), HCP (4 Nurses)	NA	NA	25	Chronic Heart Failure	MMM-M
Kane et al. 2018*	NA	Ireland	Mixed-Method; Qualitative Study; Feasibility; Interviews (Framework Analysis)	Nurse-led chronic heart failure disease management clinics in two tertiary referral centres	Patients (18), HCP (4 Nurses)	75 (8.5)	M: 11 (61)	22	Advanced heart failure: chronic heart failure: New York Heart Association functional class II-IV	HMM-M
Karamanidou et al. 2020	2019	Greece	Systematic and Mapping Review	NA	NA	NA	NA	24 primary studies	Cancer	HHH-H
Kilonzo et al. 2015	2012–2013	Ireland	Prospective Single-Arm Interventional Study	1 30-bed specialist palliative care daycare unit	Patients	M (R): 69 (47–89)	F: 19 (56)	34	Malignancy (Lung cancer, Other cancer), Noncancer (Progressive neurological diagnosis; Chronic lung diagnosis)	MLL-L
Kotronoulas et al. 2017	2014–2015	Scotland, UK	Mixed-Method; Prospective; Focus Group; Systematic Review	1 NHS board	Patients; HCP:1 gynecology CNS and 2 consultant oncologists	NA	F: 30 (100)	30	Cervical Cancer	LLM-L
Krawczyk et al. 2019a	2016	Canada	Qualitative Study (Exploratory collaborative research; participatory, focus groups, observation, interviews)	Hospital palliative consult care; acute care; large tertiary acute care hospital; large urban tertiary care centre with more than 500 beds and a dedicated 12-bed palliative unit	2 palliative nurse specialists within a larger palliative outreach consult team (POCT)	Average 66 (older adult patients (age 55+))	M: 15 (75)	20	Cancer, chronic obstructive pulmonary disease, heart failure, renal failure, cirrhosis	HMH-H
Krawczyk et al. 2019b	2014–2015	Canada	Mixed-Method; Qualitative Study; Focus Group	1 10-bed palliative unit is part of a larger 300-bed suburban acute care hospital	HCP: Nurses (*n* = 19), 1 patient care coordinator, 1 unit clerk, 1 social worker, 1 pharmacist, and 2 palliative care physicians	HCP: Median 43	F: 80%	25	Chronic life-limiting illnesses and their family members	HMM-M
Krulewitch et al. 2000	1998	US	Prospective Cohort Study; Chart Reviews	In-home assessments of community-dwelling patients and family members, adult foster care providers, or aides in residential care facilities	Patient-Caregiver Dyads	M (R): 83 (65–98)	F: 83%	156	Cognitively Impaired Older Adults: Alzheimer’s disease, vascular dementia, other dementia (e.g., MRDD/dementia or Huntington’s disease, dementia not otherwise specified	MLL-L
Krumm et al. 2014	NA	Germany	Qualitative Study; Interviews	3 Nursing homes	HCP	NA	NA	13	Dementia	HMM-M
Lee et al. 2016	2014–2015	Singapore	Pre-Post Observational Study; Retrospective Case-Notes Review Audits	Residential Hospice	HCP: Nurses	NA	NA	NA	NA	HHM-H
Lind 2018	2010–2016	Sweden	Mixed Methods; Single-Arm Interventional Study; Quantitative Descriptive Study; Explorative Design	3 Acute Care Settings in 2 Urban Hospitals in Central Sweden; 1 Palliative Care Unit in a Smaller Hospital in Central Sweden	HCP: RNs, ANs, Physicians, Healthcare Managers, Politicians	R: 65–75+	NA	Interview 1: Regional Politicians (6), Managers, HCP (5); Interview 2: HCP (37: 5 Physicians, 20 RNs, 9 ANs); Implementation: Nurse Managers (10), Internal Facilitators (15) RNs/ANs (23), Physicians (1) = 49	Pulmonary, Neurological, Gastro-Surgery	MLL-L
Lind et al. 2004	2002–2003	Sweden	Survey	Hospital-based home care clinic; advanced palliative home healthcare	Patients	NA	NA	12	Terminal cancer	MHM-M
Lind et al. 2008	2002–2003	Sweden	Qualitative Study (Cross-Case Content Analysis); Interviews; Survey; Medical Records; System Log Analysis; Qualitative Study (Descriptive and Explorative); Case Study	1 Advanced palliative home healthcare	Patients	M, Median (R): 67, 65.5 (58–79)	F: 4; M: 8	12	Palliative patients	MMM-M
Lucey et al. 2013	2007	Ireland	Mixed-Method; Survey; Focus Group	1 specialist palliative care unit; 30 bedded tertiary palliative care unit	HCP	NA	NA	20	Advanced cancer (> 90%) and where 10% have non-malignant disease	LML-L
Mahmoudi et al. 2022	NA	France	Systematic Review	NA	NA	NA	NA	66 studies (60 primary, 6 reviews)	Heart transplant recipient registry	MHH-H
Mai et al. 2018	NA	Germany	Prospective Cohort Study	Palliative care unit (PCU)	Patients	70 (12.9); R: 20–95	F: 99 (45%); M: 120 (55%)	60	End of Life: Cancer as main diagnosis (Gastrointestinal; Pulmonary; Hematological neoplasia; Urology; Gynecological; Other cancers; Dermatological cancers; Neurological diseases; Liver diseases; Cardiovascular diseases; Other noncancer diseases)	MLL-L
Martins Pereira et al. 2018	2018	NA	Umbrella Review	NA	NA	NA	NA	2 reviews	NA	HML-M
Mayahara et al. 2019	NA	US	Single-Arm Pre-Post Study; Pilot	Home Hospice (1 hospice agency)	Patient-Caregiver Dyads	M (R): Patients: 67.8 (35–96); Caregivers: 53.7 (37–69)	Patient: F: 7 (58.3); Caregiver: F: 11 (91.7)	12 dyads	Cancer, Dementia, Congestive heart failure, Other	MLL-L
Mills et al. 2008	NA	Northern Ireland, UK	Pre-Post Single-Arm Interventional Study(within an RCT); Mixed-Method (Qualitative Content analysis)	Home (Outpatient clinics at the Cancer Centre and 2 of the 4 cancer units in the country)	Patients	< 50 to 80+	M: 34; F: 23	57	Non-operable Lung Cancer (NSCLC; SCLS; Mesothelioma Unknown Primary)	MML-M
Muir et al. 2018	2007–2015	US	Prospective Single-Arm Interventional Study	Home Hospice (community-based hospice organizations)	NA	NA	NA	13,000 <	NA	MMM-M
Nair et al. 2019	NA	US	Narrative Review	NA	NA	NA	NA	NA	Kidney Disease	HHH-H
Oldenburger et al. 2020	2008–2020	NA	Topical Review	Palliative radiotherapy	NA	NA	NA	94 studies	NA	MML-M
O’Reilly et al. 2016	NA	Ireland	Pre-Post Single-Arm Interventional Study; Systematic Review	1 Specialist Palliative Care Inpatient Unit	Patients, HCP: Pre 40: medical (4), nursing (27), and therapy and social care staff (9); Post 37: medical (5), nursing (23), and therapy and social care staff (9)	68.3 (14.7)	M: 24; F: 18	42 (12 Months); 46 (6 Months); 35 (Baseline)	Malignant, Nonmalignant	MHM-M
Parker et al. 2010	NA	NA	Systematic Review	Residential Aged Care Facilities	NA	NA	NA	10 studies	NA	HHH-H
Patel et al. 2022	2019	US	Pre-Post Single-Arm Interventional Study	3 Outpatient Oncology Palliative Care Clinics: Hematology-oncology, Interprofessional, Surgical oncology	Patients	NA	NA	372 appointments	Primarily metastatic diseases	MMM-M
Pearson et al. 2007	2006	Australia	Systematic Review	NA	HCP: Occupational therapists	NA	NA	78 studies (18 reviews, 59 primary)	NA	MLL-L
Pezold et al. 2019	2017–2018	US	Pre-Post Single-Arm Interventional Study; Retrospective Chart Audits	Outpatient palliative care practice (serving patients living in their private homes or long-term care facilities)	HCP: Nurses	65 or 65+	F/M	4	NA	MHM-M
Pinto et al. 2018	2014–2015	UK	Qualitative Study; Mixed-Method; Interviews; Non-Participant Observation	9 specialist palliative care services (1 in-patient hospice, 5 hospital and 3 community teams)	38 interviewed: 7 patients, 4 family caregivers, 27 HCP: 11 doctors, 8 nurses, and 8 allied health professionals	NA	NA	38	NA	MHH-H
Potts et al. 2018	NA	NA	Systematic Review	Low-Resource Setting: homes, regional hospitals and clinics, a hospital with daycare hospice center, HIV clinics, and hospices	NA	NA	NA	18 papers	Advanced cancer, HIV/AIDs, other end-stage chronic diseases	MMM-M
Radionova et al. 2020	NA	Germany	Qualitative Study; Interviews	PC settings	HCP: 10 physicians and 9 nurses	NA	F: 14; M: 5	19 HCP	NA	HMH-H
Rauenzahn et al. 2017	2015–2016	US	Pre-Post Single-Arm Interventional Study	5 Ambulatory Oncology Clinics	Patients; HCP	NA	NA	607	Ambulatory Oncology (Breast, GI, Lung, and Head and Neck Cancers)	MLL-L
Rawlings et al. 2011	NA	Australia	Literature Review	NA	NA	NA	NA	NA	NA	LLM-L
Reynolds et al. 2019	NA	UK	Action Research; Participatory; Qualitative Study	1 Hospice	HCP: occupational therapists and physiotherapists will be referred to as AHP	NA	NA	NA	NA	MMM-M
Ruder et al. 2010	NA	NA	Literature Review	Hospice and Home Care	NA	NA	NA	NA	NA	LLL-L
Rugno et al. 2016	1999–2014	NA	Integrative Review	NA	NA	NA	NA	11 papers	NA	MML-M
Sandham et al. 2022	2017–2019	New Zealand	Mixed-Method; Interviews	1 community outpatient palliative care service	7 clinical staff members	70.9 (13.7); Median (IQR, R): 73 (20, 23–101)	404 (50.2); M: 398 (49.5); Unreported gender: 2 (0.3)	804	Oncological cancer with organ failure (heart, lung, liver, kidney), Haematological cancer, Dementia, Other neurological disease (motor neurone, multisystem atrophy, supranuclear palsy), Acute event (stroke, sepsis, abdominal aortic aneurysm)	MML-M
Sawatzky et al. 2018	NA	Canada	Qualitative Study; Focus Groups; Interviews	1 palliative home care setting and an inpatient palliative care unit	46 clinician participants: RN; medical doctors, social workers, spiritual care workers, AHPs, and licensed practical nurses, 18 patients, and 17 family caregivers	HCP: *R* = 23–63; Patients: *R* = 46–95; Family caregivers: *R* = 51–89	NA	46 clinician participants, 18 patients, and 17 family caregivers	NA	HHH-H
Schick-Makaroff et al. 2020	NA	Canada	Qualitative Study; Focus Group; Iinterviews	1 tertiary inpatient palliative care unit (10 beds) and a palliative home care setting	HCP	NA	NA	46	NA	MMM-M
Schlichter et al. 2020	2015–2017	US	Prospective Single-Arm Interventional Study	Neuroscience Intensive Care Unit	Patients	59.5 (17.6)	M: 724 (54.7)	1324	Ischemic stroke, Seizures or status epilepticus, Traumatic brain injury, Intracerebral hemorrhage, Subarachnoid hemorrhage	MML-M
Schuler et al. 2021	2018–2019	Australia	Prospective Single-Arm Intervntional Study	Palliative Radiation Therapy	Patients	Median (R): 71.5 (21–98)	F: 97 (61); M: 63 (39)	160	Lung, Prostate, Colorectal, Breast, Other	HHH-H
Schulman-Green et al. 2008	NA	US	Interviews (Telephone)	NAHC QAPI Collaborative: 9 volunteer hospice organizations: Participating hospices programs had varied characteristics in terms of size (average census 36 − 2,550); type (6 non-profit/3 for-profit; 3 HHA-based, 1 hospital-based, 1 hospital affiliated, 3 free-standing; 1 in corporate chain); experience (1 new hospice, the others established 1974–2000); and location (2 primarily rural, 3 primarily urban, 4 serving both rural and urban areas)	24 individuals representing the 9 hospices were interviewed: 6 administrators, 6 Quality Assessment (QA) directors or QA nurses, 3 performance improvement (PI) coordinators, 4 team leaders or program supervisors, 2 staff nurses, 2 hospice presidents or chief executives, and 1 chaplain	NA	NA	24	NA	MMM-M
Schulman-Green et al. 2010	2006–2007	US	Qualitative Study; Interviews (Telephone)	Hospice characteristics: Ownership type (Nonprofit, For-profit); Structure (Home health agency-based; Free-standing; Hospital-based; Hospital affiliated; Corporate chain); Location (Primarily rural area; Primarily urban area; Serve urban and rural areas)	24 individuals representing 8 hospices: 6 administrators, 6 quality assessment directors/nurses, 3 performance improvement coordinators, 4 team leaders/program supervisors, 2 staff nurses, 2 hospice presidents/chief executives, and 1 chaplain. P: 348 patients	NA	NA	24	NA	HHH-H
Schwartz et al. 2005	NA	US	Mixed-Method; Psychometric Study; Feasibility; Interviews	12: dialysis clinics (5), hospices (5), and long-term care facilities (2) in US; Implementation: 3 settings: hospice, home health, and palliative care	Patients	NA	F: 67 (40.6); M: 98 (59.4)	175	End-stage renal patients on dialysis, hospice, or long-term care patients	MML-L
Seipp et al. 2022	2018–2019	Germany	Mixed-Method; Qualitative Study; Focus Group; Field Notes	Specialised outpatient palliative care	HCP: SOPC nurse; SOPC coordinator; SOPC physician; SOPC social worker	M (R): 44.3 (37–61)	F: 10 (71.4); M: 4 (28.6)	14	NA	HHH-H
Slater et al. 2004*	NA	UK	Qualitative Study; Focus Group; Interpretive Phenomenological Approach	1 palliative day care	Patients	R: 39–81	F: 6; M: 3	9	Malignant and non-malignant disease	HMH-H
Slater et al. 2005*	NA	UK	Qualitative Study; Focus Group: Interpretive Phenomenological Approach	1 day hospice unit	4 RNs; 1 AHP; and 3 support staff	NA	NA	8	NA	MLL-L
Smith et al. 2017	NA	Australia	Mixed-Method; Retrospective Audit; Focus Group	Haemodialysis	HCP: Nurses (11)	60 or 60+ (74%)	M: 39 (69)	54	Haemodialysis; 18% dual diagnosis of malignant carcinoma including multiple myeloma, lymphoma, colon, liver carcinoma, metastatic prostate carcinoma	HMM-M
Sommerbakk et al. 2016	2012	Norway	Qualitative Study; Focus Group; Interviews; Thematic Analysis (Inductive and Theoretical)	Health care services: 2 hospitals (3 hospital services: 1 specialist PC unit, 1 geriatric/dementia unit (H-GU) in a regional university hospital, and 1 PC consult team (PCT) in a local hospital), 1 nursing home (ordinary nursing home with 1 ward specializing in dementia), 2 local medical centers (primary care service with short term in-patient care)	HCP	M (R): 51 (25–65)	F: 19; M: 1	20 HCP	NA	MLL-L
Spaner et al. 2017	2014–2016	Canada	Pre-Post Observational Study; Survey	1 palliative care unit (PCU)	HCP: RNs, registered practical nurses (RPNs), social worker, chaplain, physicians, and the patient care manager	NA	NA	20	NA	MMM-M
Stevens et al. 2005	NA	UK	Feasibility; Pilot	1 specialist cancer centre (two palliative care wards)	Patients	Median (R): 65 (25–91)	F: 23 (77); M: 7 (23)	30	Breast, Gynae, GU, Lung, Other	LML-L
Stewart et al. 2022	2020–2021	UK	Prospective Single-Arm Interventional Study	6 Different Hospital Sites (Cancer Centre (Tertiary Cancer Services))	Patients living with treatable but not curable cancer	Median (IQR): 69 (59–75)	M: 165; F: 161	326	Mixed Oncology Population: Secondary Breast, Prostate and Other	MLL-L
Stiel et al. 2012	2009	NA	Systematic Review	PC and hospice care	NA	NA	NA	725 papers	NA	MLL-L
Suri et al. 2022	2019	Canada	Implementation Research; Cross-Sectional Study	1 busy, academic, urban Canadian urban HIV clinic	Patients	NA	F: 149 (25.1); M: 445 (74.9)	600	People living with HIV (PLWH)	HHM-H
Swart et al. 2022	2017–2018	Botswana	Survey	1 hospital’s oncology Ward	Patients; HCP	Patients: 48.5 (13.9), R: 20–85; Nurses 36.5 (6.4), R: 27–55	Patients: F: 84; Nurses: F:14	124 patients; 20 nurses	Tissue-confirmed diagnosis of cancer	MHM-M
Tavares et al. 2017	2012–2014	Brazil	Observational Prospective Study; Content Analysis	1 specialist palliative care inpatient hospital unit (17 individual rooms)	Patients, Carers, HCP: 5 palliative doctors, 1 specialised palliative nurse, 1 extra nurse/shift, 1 nursing assistant for every 3–4 beds, 1 social worker, 1 psychologist, 1 chaplain	NA	F: 214; M: 186	84/317 = 401 (Pilot/Main)	Malignancy: 208; Dementia: 60; Frailty: 29; Stroke: 34; Organ failure: 40; Other: 30	MMH-M
Van Cutsem et al. 2017	2016	EU	Narrative Review; Workshop Report	NA	NA	NA	NA	NA	Colorectal Cancer	MLM-M
van den Hurk et al. 2022	NA	NA	Literature Review	Cancer Survivorship Care	NA	NA	NA	NA	Cancer	LLM-L
Viecelli et al. 2022	2019–2020	Australia	Qualitative Study; Interviews; Focus Group	4 Australian HD care units	41 participants (12 patients; 13 nephrologists, 16 dialysis nurses)	69.5 (13.41), R (39–88)	12 patients = F: 5/12 (42); M: 7/12 (58)	41	Patients receiving HD	HHM-H
Voorend et al. 2021	2017–2018	Netherlands	Qualitative Study; Focus Group	3 Dutch hospital-based study- and routine care initiatives	Patients, Caregivers, HCP: Nephrologist: 7 (28%); Geriatrician: 4 (16%); Nephrologist/geriatrician: 2 (8%); Nurse (nephrology): 3 (12%); Nurse (other): 2 (8%); Social worker: 4 (16%); Dietician: 1 (4%)	M (R): Patients: 79 (67–88); Caregivers: 60 (51–76); HCP: 48 (29–61)	Patients: M: 9 (50); Caregivers: M: 0 (0); HCP: M: 4 (16)	47	Chronic kidney disease (Geriatric)	HHH-H
Wu et al. 2022	2020–2021	Canada	Pre-Post Single-Arm Interventional Study	Home; Oncology (Radiation Therapy)	Patients (T1: 92 ± 5; T2: 100 ± 11), HCP: 2 Radiation Therapists	NA	NA	NA	Patients Receiving Radiation Therapy	LML-L

*NA* Not Available, Not Applicable, or Not Reported, *HCP* Healthcare Professionals, *M* Male, *F* Female, *M* Mean, *R* Range, *SD* Standard Deviation, *IQR* Interquartile Range; RN: Registered Nurse, *AN* Assistant Nurse, *PC* Palliative Care, *AHP* Allied Healthcare Professional, *SOPC* Specialised Outpatient Palliative Care, *HD* Hemodialysis. WoE column: *H* High, *M* Medium, *L* Low

* indicates same study from different reports

The update scoping search identified ten relevant studies [10, 134–142], which confirmed the existing findings rather than adding any new information.

**Fig. 1:**
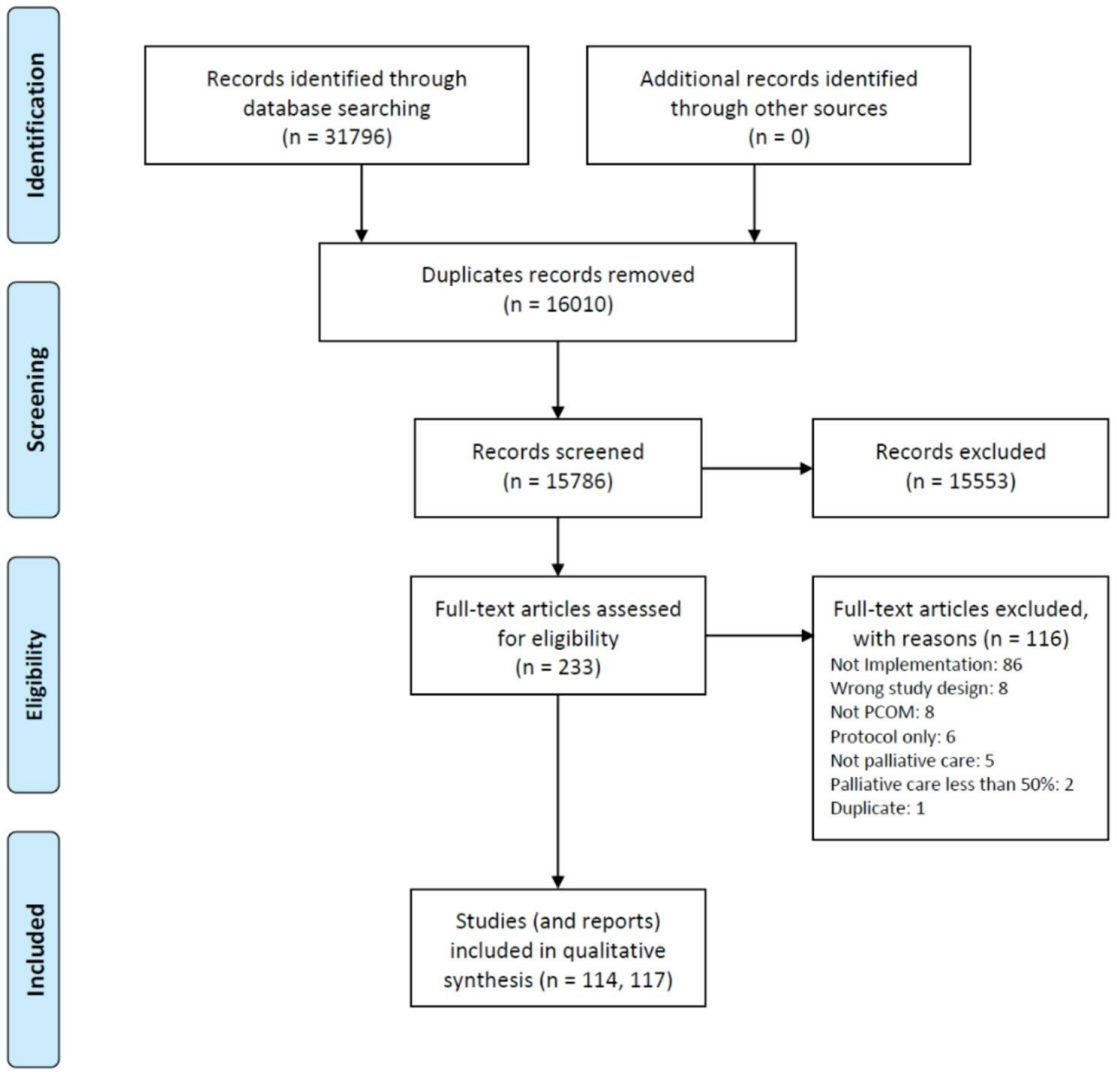
PRISMA flow chart reporting the literature search and selection of articles.

Figure 1 depicts the PRISMA flow chart reporting the literature search and selection of articles. A total of 31,796 records were identified through database searches, with no additional records retrieved from other sources. After the removal of 16,010 duplicate records, 15,786 records remained for title and abstract screening. Of these, 15,553 records were excluded as they did not meet the inclusion criteria. The remaining 233 full-text articles were assessed for eligibility. Following full-text review, 116 articles were excluded for the following reasons: not an implementation study (*n* = 86), inappropriate study design (*n* = 8), not focused on PCOMs (*n* = 8), protocol only (*n* = 6), not related to palliative care (*n* = 5), palliative care comprising less than 50% of the study sample (*n* = 2), or duplicate publication (*n* = 1). A total of 114 studies, reported across 117 articles, were included in the qualitative synthesis.

Figure 1 illustrates the geographical distribution of the included studies. Compared with the original review, which identified studies from nine countries, the updated search revealed a markedly broader international representation. The largest number of studies originated from the United States (*n* = 23), Canada (*n* = 16), and Australia (*n* = 7). Additional studies were conducted across Europe, Africa, Asia, and Oceania, reflecting the growing global engagement with PCOM implementation in palliative care. As noted in the figure legend, the appearance of several countries with a single study on the map results from a multi-country web-based survey of healthcare professionals.

**Fig. 2:**
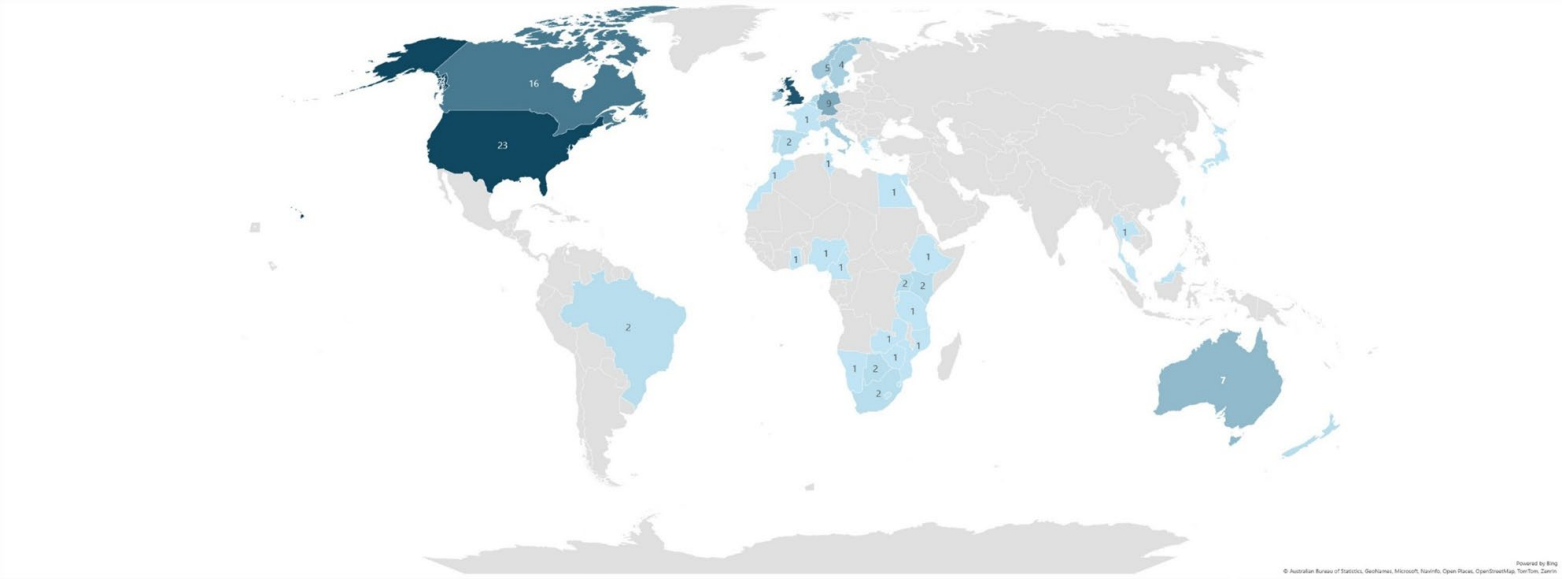
Countries of included studies.The appearance of most countries with one study on the map is because of an online multi-country web-based survey from healthcare professionals from these counties; for more details, see Table 1 of Included Studies. Data that cannot be displayed on the map are as follows: United Kingdom (21), Republic of Ireland (6), Italy (4), Netherlands (3), Belgium (2), New Zealand (2), Portugal (2), Denmark (1), Eswatini (1), Greece (1), Japan (1), Lesotho (1), Rwanda (1), Sierra Leone (1), Singapore (1), Taiwan (1), and Thailand (1)

In this updated review we present results using a two-part structure: first results are shown by objective, second, recommendations are reported using the updated Medical Research Council Framework for Developing and Evaluating Complex Interventions [20]. We use their definition of complex intervention: “*An intervention might be considered complex because of properties of the intervention itself*,* such as the number of components involved; the range of behaviours targeted; expertise and skills required by those delivering and receiving the intervention; the number of groups*,* settings*,* or levels targeted; or the permitted level of flexibility of the intervention or its components*.”

### PCOMs implemented in clinical practice

Just over 70 different PCOMs were reported to be implemented in palliative care clinical practice; half of these were only used once, therefore appearing in only one study each. The Palliative Care Outcome Scale (POS) and its family of measures were the most used family of measures with 30 mentions, followed by the Edmonton Symptom Assessment Scale (ESAS) original, revised and renal with 26 mentions. Australia is the only country that has adopted one measure, the Symptom Assessment Score (SAS) as the national standard, which is used by all specialist palliative care services in the country and the IPOS is the recommended measure for use in UK. See Appendix 3 for the full list of measures by primary studies and reviews.

### Facilitators of implementing PCOMs in clinical practice

The most notable facilitator was the integration of electronic/digital PCOMs into Information Technology (IT) systems [140–142] and routine care structures, including user experiences and user interface.

Across the included studies, several categories of facilitators for PCOM implementation were identified. Planning-related facilitators included the development of tailored implementation strategies and clear logistical preparation. At the system level, consistency with national guidelines supported adoption. Facilitators related to training and knowledge involved the provision of education to enhance clinicians’ competence in administering and using PCOM data. Organisational culture and structure facilitated implementation when teams engaged in discussions about the value of measurement and demonstrated commitment to routine use. Team-level facilitators included the presence of formally appointed implementation leads, involvement of staff in the process, and active participation from senior managers. Personal characteristics such as clinicians’ beliefs and prior experience also influenced engagement. Patient-related facilitators included coping styles that supported engagement and access to remote communication options for safety. Additional facilitators concerned effective team communication, use of PCOM data for continuous service improvement, and allocation of sufficient time for implementation activities. Resource-related facilitators included supportive infrastructure, readiness for change, adequate staffing, shorter completion times—particularly for electronic formats—and potential cost efficiencies associated with digital integration. Factors related to the PCOMs themselves, such as their ability to support patient expression, as well as flexible timing of administration, user-friendly equipment, and appropriate data security and equity measures, also supported successful implementation.

See Appendix 4 for the full list of categories and sub-categories of facilitators.

### Barriers to implementing patient-centred outcome measures in clinical practice

The main barriers are mainly related to healthcare professionals, including: resistance to change, negative attitudes towards changing routine, scepticism regarding the necessity and usability of data collected, concerns regarding privacy and confidentiality, and belief that data collection is a burden to patients but also for themselves, given high workload:

Regarding resources, lack of training, time constraints, access to the tool and equipment to support electronic tools, were widely mentioned, as were unwell and complex patients who might not be able to fill the measures or have a rapidly changing condition that they felt the measure might not reflect:

Regarding the measures themselves, there were concerns relating to complexity, adaptability, interpretation, individualisation, comprehensibility, timing of use, numerical scoring, acceptability, and lack of consensus on what tool to use and their psychometric properties. See Appendix 5 for the full list of categories and sub-categories of barriers.

### Lessons learned on implementing PCOMs in clinical practice

Implementing a PCOM in palliative care clinical practice is a complex intervention which needs planning, assessment and fine tuning throughout its course at different levels and with all involved [85]. We identified the following, mainly taken from the discussion section of included studies.

#### Guidance

Step by step guides specifically designed to implement PROMs in clinical practice [22, 25].

#### Clinical Staff

Important to integrate nursing staff in the implementation process early with specific training and feedback. Consider individual attitudes and motivation to use new evidence-based practice. Changes that require clinicians to modify the interpersonal aspects of their care, or that are more complex, may be perceived as more difficult to implement [2, 21, 23, 47, 54, 93, 128].

#### Measure

Measures must be psychometrically sound and comparable [2, 34, 45, 47, 59].

#### Measurement frequency

When managing poorly controlled symptoms, measurement is needed multiple times a day [2, 22, 25, 41, 47, 75].

#### Stability of measures

Once a measure is introduced, there needs to be a commitment for it to remain in place for a long period. Changing versions and measures should not be undertaken lightly as every change requires changes in IT and staff training and comes at a considerable cost [36].

#### Setting

The context in which measures are introduced and used is important. There are currently no palliative care measures for Intensive Care Unit (ICU) and paediatric palliative care settings. It has been suggested that the use of PCOMs in the palliative radiotherapy settings may not be possible, due to the large variety in pathologies, radiation schemes and treatment indications [25, 34, 45, 46, 56, 61, 65, 93, 106, 115].

#### Implementation

Implementation is a time-consuming and long-term process and needs continuous attention and flexibility. Using the principles of change management, factors other than those relating to individual professionals are important, and greater use of theories of change may help to explain whether change is possible. Using PCOMs in palliative care clinical practice is a complex intervention; organisations’ aims must align with the effort to aid their clinical teams, with quality of care at the forefront as the rationale for implementing PCOMs [22, 38, 39, 41, 43, 44, 47, 85, 115, 119, 128].

#### Feedback

Across the studies reviewed, several forms of PCOMs feedback were identified as important for supporting care processes. These included individual patient-level feedback provided to clinicians, often in graphical or summary form to highlight symptom severity or change over time; feedback delivered directly to patients and family caregivers to support shared understanding; and aggregated feedback at team or service level used for monitoring quality and informing improvement activities. Timely and repeated feedback, particularly when integrated into electronic systems or displayed through dashboards, was also reported as facilitating clinical use. Together, these forms of objective, structured feedback were consistently valued by patients, family caregivers, and health professionals, even when patients’ health was deteriorating [7, 13, 30, 36, 39, 47, 54].

#### Patients

It is important to distinguish between patient burden and patients being too unwell to complete measures. PCOMs enable systematic symptom monitoring, and when ePROs trigger clinical alerts, they prompt timely clinician response to urgent patient needs. Patients who trigger electronic clinical alerts tend to be younger and more recently diagnosed, to have greater comorbidities, and to be from racial/ethnic minority groups. Clinicians and patients perceive that, when used in first assessments, individualised PCOMs supported relationship-building because they enable patients to ‘tell their story’: however, if the clinicians feel they cannot resolve an issue, there is a tendency to disregard and close the conversation [11–13, 39, 54, 62, 63, 75].

#### Technology

Future digital health approaches in palliative care require understanding patients’ needs, and PCOMs help capture these systematically to guide timely, personalized clinical responses [21, 23, 30, 34, 93, 95]. See Appendix 6 for full list.

### Implementation models used when implementing PCOMs

A total of 18 models, frameworks and theories were identified as used in just 25 of included studies, most commonly Plan-Do-Study-Act (PDSA) [143], Promoting Action on Research Implementation in Health Services Framework (PARIHS) [144, 145], and Consolidated Framework for Implementation Research (CFIR) [133] PDSA is an iterative, four-stage model that asks three questions: what are we trying to accomplish, how will we know that a change is an improvement and what changes can we make that will result in an improvement? PARIHS identifies three key components of the implementation process: evidence, context, and facilitation. Implementation is more likely to occur when evidence is scientifically robust, aligns with practitioner and patient beliefs and local experience, the context is receptive to change, and there is appropriate input from internal and external facilitators. CFIR, updated in 2022, has five domains to guide systematic assessment of potential barriers and facilitators, tailor implementation strategies and needed adaptations, and explain outcomes (individuals, inner setting, outer setting, implementation process and innovation/change to be implemented) [146].

See Appendix 7 for a complete list of the implementation models and frameworks identified.

### Implementation outcomes measured, and how, when implementing PCOMs

Sixty-three included studies mentioned at least one implementation outcome and most studies reported only on feasibility of implementing a PCOM or e-PCOM in clinical practice and the acceptability to patients and healthcare professionals of the measure used.

Only two studies mentioned appropriateness: one considered three implementation outcomes [117]. It was concluded that by documenting evidence of pain assessment and screening for spiritual distress, the number of referrals improved and was maintained 12 months and more clinicians agreed that palliative care domains were comprehensively assessed post-intervention [117]; the other mapped to NPT constructs and the Context–Mechanism–Outcome framework. The study captured acceptability (professionals’ perceived benefits and burdens), appropriateness (fit with workflow and electronic systems), feasibility (practicality given staffing, routines, and IT capacity), adoption (extent and consistency of use), fidelity (variation in scoring and assessment practices), and initial indications of sustainability (whether teams continued after the study). These outcomes emerged entirely from thematic analysis of interview data rather than quantitative measurement [142].

## Financial costs of implementing PCOMs

No included studies reported on the costs of implementation. Some provided suggestions in relation to costs, for example suggesting that the way to rationalise PCOMs implementation is for these to replace routine procedures [21, 28] or as a complement to existing practices without requiring additional resources [73]. Others suggested that implementation would be costly in the short-term but cost-saving in the long term by reducing the workload [21, 30, 117]. One study observed that teams allocated work hours to using PCOMs and occasionally accepted overtime [142].

Studies suggested that the possible costs of implementation include: cost of the PCOMs [21, 106]; cost of healthcare staff time and capacity [41]; cost of creating, maintaining and updating electronic systems and integration into existing IT systems [41, 67, 117, 130]; costs to healthcare system of reimbursement and pay-for-performance incentives [30, 41, 92, 121] setting-dependent costs including models of care, clinical competencies and resourcing [36]; unavailability of reimbursement/insurance plans to cover staff time in the US; [41] lack of funding in charitable organisations [106]; and costs associated with the outcomes of implementation including resources to respond to patients’ issues identified [8, 41]. Some solutions were proposed to save costs [8, 21, 41, 44, 50, 102, 117]. See Appendix 8 for a full list of facilitators and barriers regarding financial costs.

### Updated recommendations for PCOMs implementation

Based on the new evidence synthesis, we have updated recommendations, and reiterated those from our previous review, by mapping them on the updated MRC Framework for Developing and Evaluating Complex Interventions [20]. Table 2 presents a summarised description of the recommendations mapped on the MRC Framework in the form of actions. Below, we describe the main considerations arising from those actions.

**Table 2:** Recommendations per phases and respective core elements following the latest framework for developing and evaluating complex interventions by the medical research Council

PHASES CORE ELEMENTS	Identify the intervention: Implementing PCOM in palliative care clinical practice	Feasibility testing	Evaluation	Implementation
**Context**	- Use “User’s Guide for Implementing Patient-Reported Outcomes Assessment in Clinical Practice” to help with discussions around the advantages and disadvantages of options for key design issues such as selecting questionnaires; determining the frequency with which the questionnaires are administered; determining when, where, how, and to whom the results will be presented; planning the response to patient needs identified by the PROs; and evaluating the use and benefit of PROs in a particular setting.^30^ **- There is an urgent need to develop a palliative care PROM for the ICU setting**,** as is a paediatric palliative care PROM.**^**61**^ **- Consider the following implementation steps: (I) selection of outcomes of interest; (II) selection of outcome measure(s); (III) education about the measure and use of results; (IV) selection of one coordinator/facilitator; (V) who applies the measure and its periodicity. Implementation is time consuming and a long-lasting process needing continuous attention**,** never seems to be completed and touches on principles of change management.**^**27**^ **- Be aware of issues for a speciality when selecting and implementing outcome measures in practice: different specialities have different challenges and concerns** ^**111**^ **- Challenges for using outcome measures are different in different settings** ^**111**^ **- Bespoke measures important to patients and their families must be collected prospectively and they cannot be derived from clinical records** ^**41**^ **- By controlling for patients’ overall physical status (which is the major predictor of resource utilisation at the end of life) in the comparisons made**,** residual variations are largely going to be due to variations between services: models of care**,** clinical competencies**,** resourcing or combinations of these factors. This has allowed a process of embedding quality systematically across a whole sector of the health system relatively quickly. Developing a culture of rapid evaluation and re-evaluation after adjusting local models of clinical care delivery is an exciting development within hospice/palliative care.**^**41**^ **- Understand the health care organization context**,** including alignment with priorities and purposes regarding current and past use of QOL assessment instruments in the organization.**^**115**^ **- To achieve significant and sustained improvements in patient care in response to the feedback of performance data**,** it is imperative to use system and organisation-wide strategies**^**59**^ **- Use measures that are suited to the clinical task being delivered and suited to the aims of your clinical work and the population you work with. They differ in the domains and dimensions they measure**,** and in their length**,** measurement window**,** accessibility and cost**^**2**^	- Initial meeting to explore feasibility of implementation of the measure: planning and evaluating are essential; a coordinator is identified to undertake overall responsibility - Appraise characteristics and resources of the setting and the requirements of the proposed innovation: that is, it is necessary to attune the implementation protocol to the needs of the specific setting and think about good documentation: will allow staff to take ownership and understand the benefits of using the chosen measure, which needs to be adaptable to local circumstances - Education and training sessions: consider your setting and organisational needs, when planning, that is, timing of sessions **- It is vital to assess organizational context when implementing an evidence-based complex intervention and having a plan in place to ensure practitioners remain motivated to use the new intervention** ^**43**^	- Assess if collation of data generated using the measure allows continuous, accurate collection of information, which should reflect the activity of the palliative care team. It should also identify areas of potential future development - Assess if changes will be made in practice based on the results of implementing and using the measure	- Maintain strategies of reminders to incorporate the use of the measures in clinical practice, for example, daily assessment easily fits with daily routine and does not take additional time, that is, if it is incorporated with moment of control of vital signs: better insight into patients’ situation **- Need for better understanding of how institutions can join efforts to assist clinicians and services to implement PCOMs in PC clinical practice** ^**133**^ **- In kidney disease care**,** setting and time of electronic collection: clinic intake room before appointment (CKD/transplant); dialysis facility during dialysis treatment; home during home dialysis session; home between clinic appointments or dialysis sessions (via ecological momentary assessment)**^**98**^ **- Storage: Integration into electronic medical record with password-protected access; Interpretation; Incorporation into risk prediction models; Comparison with national benchmarks** ^**98**^
**Programme Theory**	- Physical functioning needs to be clearly defined and conceptualized, and a consensus on terminology and which aspects that are relevant for palliative care is highly warranted.^76^ **-** M**ore systematic use of theories in planning and evaluating quality improvement interventions in clinical practice theories may help to explain whether change is possible**^**27**^	**-** C**onsider theoretical aspects in the development of documentation systems or measurement instruments**,** to avoid different measurement levels for sub-items.**^**52**^ **- Action research provides structure to involve healthcare professionals in introducing outcome measures within a practice setting.** ^**111**^	**-** I**ndividuals within an organization learn within the social context of other learners. People are “not passive recipients of innovations” but rather they seek to evaluate them and communicate about them.**^**43**^	**- Implementation may be more successful for services that offer regular opportunities to use the intervention in practice**,** have sufficient levels of facilitators**,** stimulate more staff discussion**,** and encourage maintenance of positive motivation.**^**43**^
**Stakeholders’ engagement**	**- Meaningful outcomes can be routinely collected in hospice/palliative care and**,** that by providing a feedback loop and service to service benchmarking**,** patient-focused improvements can be delivered.**^**41**^ **- Involve palliative patients in different settings in studies testing electronic PRO data assessments** ^**39**^	- Establish clear boundaries at the outset to avoid unrealistic expectations from all actors involved - Visualisation of results should allow easy and quick interpretation by health-care providers, patients and their families - Consider investing in training of clinical key staff: improves data quality and demonstrates that data can be used (a) on a daily basis as part of clinical practice and (b) to manage and improve services, that is, data can be used in case conferencing and interdisciplinary team meetings and at staff handovers for patient care, discharge planning and discharge and transfer of patients as well as audit for quality assurance - Organisational support is needed to maximise the tool’s impact: mechanisms for sharing the information with caregivers need to be developed - Initiate implementation in stages to improve acceptability - Coordinator maintains good relationships with all involved by having both an awareness of the extra time and effort needed to implement new outcome measures and providing the appropriate resources and practical support to use the measures and carry out data analysis. Cascade management style is adopted - Integrating nursing staff into the implementation process at an early stage. This integration includes offering specific training in the use of the documentation interface in addition to the possibility of providing feedback and adapting the measurement system to foster its ease-of-use.^52^ **- Changing the practice is complex: provide enough time**,** continuing support**,** and quality in the methodologies of change enacted.**^**45**^	- Assess if practice improved initially as a result of just ‘planting the seed’ of the patient’s needs management. Continue to encourage its use, which will improve confidence with the tool - Assess if there is a benefit to both patient and practitioner in achieving better outcomes, improved concordance and potentially reducing the cost and effort of that management - Collected items are reviewed with the aim of being clinically relevant and not burdensome to collect **- PCOMs reflect the excellence of the health service delivered and to use them in a continuous base for quality improvement and programme certification also allows for decision and policy makers to have more concrete evidence to support and make the best decisions** ^**133**^	- Education and motivation of patients could improve compliance - Timely feedback of results is always done - Persistence and encouragement by the coordinator are necessary to ensure the implementation is successful and that communication among all actors involved is clear - Coordinator investigates all complaints/issues and addresses them immediately, at the time of the complaint/issue (negative views from both patients and professionals tend to be at the outset of implementation when they have not familiarised with the measure) - Every member of the team who should be using the outcome measures does so **-** I**n kidney disease care**,** method and mode of collection: self-administered online surveys via tablet computer or smartphone; nurse-administered online surveys via tablet computer**^**98**^ **- Regular sharing of results with patients**,** caregivers**,** and clinicians; Action; Targeted**,** individualized treatments based on results (referral to psychologist**,** change in dialysis prescription)**^**98**^ **- Objective feedback from PCOMs is paramount: patients**,** family caregivers and health professionals value it**,** even in the face of deteriorating health**^**13**^ **- When used in first assessments**,** individualised PROMs support relationship-building: they enable the patient to ‘tell their story’.**^**59**^ **- PCOMs facilitate discussions between patients and nurses about care needs. Patients feel empowered.** ^**68**^
**Key uncertainties**	**-From an ethical perspective**,** consider that by either excluding patients with dementia or cognitive impairment from their study samples or by performing a proxy-assessment of needs and outcomes**,** some vulnerable patients are at risk of developing further vulnerabilities**^**94**^ **-Several factors may influence the success of the implementation of electronic PRO**,** such as cultural and socioeconomic**,** e-health literacy and care setting (inpatient vs. outpatient)**,** because patients’ goals and care needs often differ**^**39**^ **-A clear guideline on the use of PROMs in the palliative radiotherapy setting may be impossible**,** due to the large variety in pathologies**,** radiation schemes and treatment indications.**^**100**^ **- There is tension between PROMs as a QI strategy versus their use in the care of individual patients; some PROMs that clinicians find useful in assessing patients**,** such as individualised measures**,** are not useful as indicators of service quality.**^**59**^	**-Potential risk of harm of using a measure in routine care. There are risks of inaccurate assessment either due to poor assessment or lack of measurement reliability** ^**50**^ **-Awareness of the potential of detracting from caring responsibilities in under-resourced care settings.** ^**50**^	- Assess if there is a benefit to patient in achieving better outcomes	**- There is an important distinction between patient burden and patients being too unwell to complete outcome measures – these are often conflated.** ^**13**^ **- Recognise that patients**,** families and professionals may have differing views about the advantages and disadvantages of using outcome measures**,** particularly in relation to feelings of reassurance and burden.**^**13**^ **- Whether PROMs support or constrain patients in sharing or raising issues with clinicians depends on the structure of the PROM**,** e.g.: standardised PROMs were useful for those patients who preferred not to talk about personal or sensitive issues**,** helping them to share information.**^**59**^
**Intervention** **refinement**	**- To maintain the possibility of evaluating a proxy-based measurement system with respect to its psychometric properties**,** it is highly recommended to add an empirically validated instrument for data collection.**^**52**^ **- International developments can be unlocked by providing controlled access to ePRO data**,** algorithms**,** and models. The use of ePROs is instrumental in driving innovation in the eHealth era. For symptom management**,** it is shifting care from reactive to proactive and preventive by integrating predictive devices into the patient’s daily life.**^**135**^	- Discussions in this step should include assessment of current data collected, how data are collected, what new data items are to be extracted, how data can be extracted and provided to who will analyse it, and, which measure(s) to use, especially due to the high number of existing measures and the fact that many are used in research or were used in one study only. A measure broadly used will allow for comparisons. The aim should be to embed the collection of standardised clinical assessment and other clinically relevant data into daily clinical practice with a view to improving clinical care - Consider implementation strategies: consistency is paramount. Frequency of use of the measure; burden of completion on patient; measures that can be analysed using existing resources; a measure that is easy and quick to use; establish link with a research/audit group **-ePRO survey can be used even in patients with a high burden of disease as well as in older patients** ^**58**^	- Collected items are reviewed with the aim of being clinically relevant and not burdensome to collect - Assess if the measure generated valuable information, without an increase in paperwork, potentially freeing up some time to deliver more patient-centred care - Continue to refine the process to make it more understandable and acceptable to patients and caregivers - Assessing and improving documentation will potentially improve practice and quality of care by highlighting needs **- Seek relevant patient and staff feedback on observing and implementing the measures considering lessons learned in the process** ^**44**^	- Data that are more time consuming to collect but important for quality reporting are collected periodically, rather than, for example, daily - Space and time are created to discuss how implementation is going: problems and benefits of using outcome measures are easily and rapidly noticeable (what is working and what is not) - Interpretation of results is used in practice **- Symptom burden is subject to frequent fluctuations in its intensity within a day: a high-frequency measurement approach of symptom burden data is highly recommended** ^**52**^ **- Initial decisions taken for implementation are not final and once the protocol is put in practice**,** several processes will need to change across time**^**133**^ **- It is imperative to find strategies to widen participation using electronic measures across patient groups. On-site completion is achievable with limited staff support**,** but remote electronic completion requires further work to improve systems and acceptability for patients.**^**26**^ **- Conceptualize and measure implementation outcomes to determine the effectiveness of implementation strategies. Need to measure implementation outcomes systematically.** ^**13**^ **Regarding dementia patients in care homes: improved observation and awareness of residents**,** collaborative assessment**,** comprehensive ‘picture of the person’**,** systematic record keeping**,** improved review and monitoring**,** care planning and changes to care provision**,** and facilitated multi-agency communication. Potential benefit included improved symptom management**,** improved comprehensive care**,** and increased family empowerment and engagement.**^**50**^ **- Standardisation and technologization of medicine cannot and must not replace a personal conversation between health care professionals and patients.** ^**108**^ **Specific to dementia**
**Economic considerations**	**- There is a disconnection between the development of PROMs (and PREMs) and the implementation of value-based health-care (VBHC)**,** a continuous implementation process still in its infancy**^**49**^	- Consider establishing a program evaluation system for the first month of each phase as well as every 6 months during the first year of implementation. Should include feedback from patients, staff members, physicians, nurses (and ITS team if applicable) as well as cost savings to the setting - Investment in computerised systems for quick data entry and analysis should be considered. Ways of visualising, storing, retrieving and backing up the data should be discussed **- Use of PCOM in PC clinical practice**,** take into account individual needs**,** values and patient and families’ resources**,** which adds a much needed measure to help guide improvement of quality of care provided.**^**133**^ **- Capability measures are appropriate for use in economic evaluations of interventions at end of life** ^**38**^		

New recommendations are in **bold**

#### Intervention development/identification

Implementation of a PCOM in palliative care can occur in a range of clinical contexts including hospital, home, hospice, or care home. These contexts have different rules, different resources available, and different support from managers and different outcomes the team wish to measure [2, 7, 34, 36, 38, 85, 106, 110]. Clinicians may not have experience or training in using PCOMs [21, 22, 38, 47, 61, 128]. Patient populations will differ, being more or less complex and in a more or less advanced stage of illness [13, 30, 46, 47, 85, 103, 106]. Very ill patients may have missing data [2, 46, 54, 85], in which case a PCOM which allows for proxy ratings may be appropriate [2, 34]. These factors will all influence the measure(s) selected [2, 46, 54, 75, 85].

The use of theory to underpin implementation models is generally considered beneficial, as it provides a structured foundation for understanding and guiding the implementation process. From the theories available in the literature, it will be important to select one which is appropriate for the clinical context in which the implementation will be conducted [22, 38, 47, 106, 121].

Engaging stakeholders from the initial planning phase is vital, including: patients, families and other informal caregivers, healthcare professionals from all the teams involved, managers, policy makers, and decision makers [13, 22, 38, 40, 47, 54, 106, 110, 128].

Identification of key uncertainties, including the adjustments to be made during the different steps is key [2, 21, 25, 38, 47, 54, 85, 128]. Consideration needs to be given to: when will patients be asked to complete the measure, who will ask the patient, will patients choose paper or electronic formats, whether a family or staff member will be able to act as a proxy if the patient is unable to complete the measure, will the institution provide IT resources for optimal use of the PCOM information, when will the feedback happen to healthcare professionals and patients, how will responses be used to support or improve patient care (and/or quality improvement), when will the team meet to discuss implementation [7, 13, 30, 36, 39, 47, 54]. Other uncertainties will arise as the implementation moves forward and clinicians become more familiar with the new routine [2, 8, 30, 54, 63, 74, 75, 85, 128].

Refining of the intervention can only take place after the team has met to review how the implementation is going and changes needed [40, 85, 128]. Consideration needs to be given in the planning phase to minimising missing data (this appears to improve with electronic data capture), how often will clinicians use the results and how will this be reported, how engaged are all members of the clinical team in the new routine, and other ways to improve the implementation [2, 22, 25, 36, 38, 54].

Economic considerations must be contemplated from the outset [2, 33, 44, 121]: for example, is there additional resource to support the implementation or will this be within current resources and workforce time available [8, 30, 36, 41, 64, 67, 73, 117]; what IT and administrative support will be available initially and long term to store, synthesise and present data from the measure to clinicians [50, 79, 92, 106, 130, 132].

#### Feasibility

Feasibility testing, against predefined progression criteria [2, 34, 45], assesses feasibility and acceptability of implementing the PCOM(s) in the clinical setting concerned [2, 13, 34, 47]. This allows decisions to be made about progression to the next stage. Consideration of the optimal way to integrate the use of the measures and the use of their results in clinical practice [2, 54, 85] requires team leads to all be on board and meet periodically to discuss the six core elements as the phase progresses [2, 8, 13, 45, 54, 75, 128].

Context includes the setting where care is delivered, the institutional culture, the staff members involved, the physical setting in which patients will fill in the measure and when they will do so [9, 38, 54, 109, 110, 128]. The theory employed needs review regarding how well it fits with the implementation setting [38, 57, 83, 101]. Stakeholder engagement needs to involve patients and family members, clinicians, decision makers and policy makers [2, 13, 22, 25, 36, 38, 54], in order to understand if it is feasible to implement the selected PCOM, identify key uncertainties and how they will be explored and resolved [22, 47, 128]. One important issue raised in the included papers was the course of action when a patient reports a very high score for one or more items, when that indicates the worst possible state [2, 13, 22, 25, 34, 36, 39, 47, 54, 63, 69, 74, 93, 106, 107, 109, 128].

Regarding economic considerations, it is important to understand how much time the clinical, administrative and IT teams will spend on the implementation and whether that will be resourced [2, 22].

#### Evaluation

The evaluation phase investigates whether and how the intervention works, the usefulness of the information gained, how it interacts with the clinical context and contributes to improved outcomes for patient and families and system change [22, 39–41, 45, 54, 75, 85, 93, 121, 128]. Strong networking relationships are likely to be established with formal and informal sectors outside of the organisation [38, 40, 41, 54, 85], including identification of outcomes which capture changes to the wider healthcare system [2, 13, 21, 36, 41, 45, 47, 54, 72]. When refining the theory, it is important to consider the interpretation of data and how it will have an impact on clinical processes and outcomes [38, 57, 83, 101]. If the results of the PCOMs are being used, that should lead to improved levels and quality of care and improved patient and family outcomes [2, 4, 22, 36, 54, 72, 82, 128]. When healthcare professionals are trained to interpret and act on PCOM feedback, care becomes more responsive to patients’ changing needs. Furthermore, using PCOMs systematically allows for benchmarking and continuous quality improvement at an organizational level, creating a feedback loop that fosters accountability and patient-centered service development. These mechanisms can ultimately contribute to better alignment of care with patient goals and improved experiences at the end of life. This underscores the importance of systematically assessing not only whether PCOMs are implemented, but also how they influence clinical practice and patient trajectories. Evaluation should explore the mechanisms through which PCOMs inform care—such as changes in clinical decision-making, timeliness of interventions, or communication with families—as well as measurable improvements in health outcomes and satisfaction. Additionally, the evaluation phase provides an opportunity to examine contextual factors, such as staff engagement or organizational readiness, that may mediate or moderate the impact of PCOM use. Evaluation should primarily focus on the performance and sustainability of measures within routine, long-term practice settings, rather than being limited to isolated or short-term studies [26]. The best example are the PCOC measures which have been collected in Australia continuously for 20 years.

#### Implementation

After developing and planning the implementation, piloting it, assessing how that first stage went and making changes, it is expected that there will be a “second try” that is better adapted to the reality of each clinical team, as an ongoing process. The six core elements will be unique to each clinical team and dependent on the previous work carried out. If the main issues which arose in the pilot phase were addressed in the evaluation phase, the implementation should run more smoothly with less issues to resolve, as illustrated in the Tavares AP et al. study describing all four steps of implementing a PCOM in an inpatient palliative care service [128].

By rigorously evaluating these components, we can determine whether and how PCOMs contribute to better patient-centred outcomes and generate transferable insights to guide future implementation and scale-up.

## Discussion

Despite a decade of expansion in the literature (from 26 studies to 114), the core facilitators, barriers, and lessons learned in implementing PCOMs in palliative care clinical practice remain largely consistent. As in the original review, the updated synthesis confirms that successful implementation still depends heavily on organisational planning, the presence of a coordinator, staff training, perceived clinical relevance of the measures, and supportive team culture. Likewise, persistent barriers—time constraints, staff workload, lack of training, concerns about burden, and clinicians’ beliefs or reluctance—remain fundamentally unchanged. However, two important differences emerge. First, the updated review identifies digital integration of PCOMs into IT systems as a major new facilitator, reflecting technological developments absent from the earlier evidence base (e.g., user-friendly e-health interfaces and digital workflows). Second, the contemporary literature shows a broader international reach and introduces new domains—particularly implementation models, implementation outcomes, and cost considerations—which were not addressed in 2014. Despite these additions, the essential message of both reviews is consistent: implementing PCOMs remains a complex intervention requiring ongoing planning, adaptation, and engagement across all levels of palliative care practice..

### PCOMs implemented in clinical practice

The PCOMs most widely used continue to be the POS and the ESAS families of measures. There are a plethora of outcome measures which appeared only once in this review.

### Facilitators of implementing PCOMs in clinical practice

Despite a large increase in the volume of literature on PCOMs implementation since the original review (117 vs. 33 studies), in a lot more countries, illustrating how the field has expanded internationally since 2012, evidence on facilitators and barriers to implementation of PCOMs is largely unchanged since 2014. An exception to this is increased focus on the integration of electronic/digital PCOMs into IT systems and routine care structures, including user experiences and user interface as a facilitator to implementation. Recent studies conducted in specialist palliative home care similarly report that implementing ePROMs introduces new layers of complexity, particularly around digital workflows, data privacy, and professional workload [140, 141].

### Barriers to implementing patient-centred outcome measures in clinical practice

Organisational culture and privacy/confidentiality issues are more evident as barriers in this updated review, as healthcare professionals continue to raise significant concerns regarding the wider adoption of digital PCOM systems These concerns often relate to the security of electronic data, the potential for unauthorised access, and uncertainty about how sensitive patient information may be stored, shared, and protected within organisational systems. Addressing these issues through robust data-governance practices and clear communication about safeguards is essential to building clinicians’ trust and ensuring confident, sustained engagement with PCOMs in routine practice.

### Lessons learned on implementing PCOMs in clinical practice

The implementation model presented in Fig. 1 of the 2014 review and the updated MRC Framework show clear conceptual alignment, with both depicting implementation as an iterative, phased process shaped by organisational context, stakeholder engagement, and continuous refinement. Each highlights the importance of early preparation, coordinated planning, staff involvement, and mechanisms for feedback and adaptation. Although developed a decade apart, both frameworks recognise implementation as dynamic rather than linear, influenced by interactions across individual, team, and system levels.

Important differences, however, reflect the broader evolution of implementation science. The 2014 model is practice-oriented and tailored to the specific realities of introducing PROMs/PCOMs into palliative care, emphasising operational steps, behaviour change processes, and the practical barriers and facilitators encountered in clinical settings. In contrast, the updated MRC Framework provides a more comprehensive and theoretically grounded guide for developing, evaluating, and implementing any complex intervention. It extends beyond workflow considerations to include programme theory, feasibility, uncertainties, and economic dimensions. Thus, while the earlier model offers a pragmatic roadmap rooted in clinical experience, the MRC Framework provides a wider methodological structure aimed at ensuring rigour and scalability across diverse contexts.

### Implementation models used when implementing PCOMs

One of our new objectives was to identify the implementation models used, which the MRC guidance considers a core element for each phase. “*Frameworks provide a base set of concepts*,* terms*,* and definitions by which to articulate dynamic complex contexts and develop much needed measures of context*.” [146, 147, 148]. Clinical teams need to recognise the ongoing iterative nature of implementation, using theory to structure the main actions and changes that need to take place when implementing PCOMs. This aligns with newer work drawing on Normalisation Process Theory to understand how PCOMs are operationalised within home care teams and the factors influencing sustained use [142].

### Implementation outcomes measured, and how, when implementing PCOMs

Although most included studies mention implementation outcomes, the majority only mention one or two out of the eight developed by Proctor et al. (acceptability, fidelity, feasibility, adoption, appropriateness, penetration, sustainability, and cost) [149]. Future studies need to take into consideration a greater number of implementation outcomes. Appendix 9 provides a quick overview for clinical teams regarding objectives three to seven’s results.

### Financial costs of implementing PCOMs

Many healthcare systems focus more on organizational efficiency and cost containment than promoting person-centred care, ascribing value to “bureaucratic models” of task-based care [79]. However, “the rationale for implementing pain assessment systems in palliative care should be based on quality of care rather than reduction of visits” [82]. Stiel et al. (2012) [125] list eight outcome assessments related to the economy of the healthcare system in relation to palliative care: costs, length of stay, reasons for admission, quality of care, discharge disposition, number of admissions, funding of palliative care, and facility size. Clinicians and researchers could usefully consider include all of these to provide clearer and in-depth information on costs.

### Updated recommendations for PCOMs implementation

The main clinical and research implications of our findings highlight the central importance of staff engagement and training staff in PCOM tools, communication strategies, and cultural competence. The literature suggests that there is better continuity and coordination of care, when PCOMs are used longitudinally, improving shared decision making by sharing PCOMs results between patients, families and clinical teams. Simple cross-sectional studies using one measure do not test sustainability or equity, since they do not allow for comparisons at patient, team or policy maker levels. Future research needs to focus on implementation outcomes because understanding how interventions perform in real-world settings is essential for their success, as is ensuring that innovations like PCOMs are effectively integrated and lead to meaningful improvements in patient care. Costs and longitudinal study approaches also need to be considered.

Finally, proxy-rated PCOMs remain essential in palliative care, particularly when patients are unable to self-report. While tools such as the IPOS have undergone robust validation in proxy-report formats, the development of novel proxy-specific methodologies remains limited, with recent research largely focused on validation rather than advancing implementation strategies.

### Strengths and limitations

A major strength of this updated and expanded review is the addition of more objectives to those of our previous review, including examination of frameworks and models of implementation, which provide a base set of concepts, terms, and definitions. Another strength lies in its rigorous and transparent methodology, which was developed and reported in strict accordance with established international guidelines, enabling full replicability. A potential limitation of this study is the heterogeneity of the included studies. However, we feel it was appropriately addressed through the use of a rigorous narrative synthesis, which allowed for systematic comparison, contextual interpretation, and transparent reporting of patterns across diverse study designs and settings. h [10, 134–142].

## Conclusion

This updated systematic review synthesises contemporary evidence on the implementation of patient-centred outcome measures (PCOMs) in palliative care clinical practice. The findings confirm that the POS and ESAS families of measures remain the most commonly implemented PCOMs, while a wide range of other measures are used inconsistently across settings. Despite a substantial expansion of the evidence base since the previous review, the core facilitators and barriers to implementation remain largely unchanged. Successful implementation continues to depend on organisational support, staff engagement and training, and perceived clinical relevance, whereas time pressures, workload, and clinicians’ beliefs persist as key barriers. The integration of electronic PCOMs represents an important recent facilitator but also introduces new challenges related to workflow, data governance, and privacy.

Implementation of PCOMs is consistently characterised as a complex, iterative process. Although a growing number of studies draw on implementation models and frameworks, their application remains uneven and often implicit. Assessment of implementation outcomes is limited, with most studies focusing narrowly on feasibility and acceptability, and there is a complete absence of empirical evidence on the costs of implementation.

Future research should prioritise theory-informed, longitudinal implementation studies that systematically assess a broader range of implementation outcomes, incorporate robust economic evaluations, and examine sustainability, equity, and scale-up across diverse palliative care contexts. Addressing these gaps is essential to support the routine and sustainable use of PCOMs to improve patient- and family-centred palliative care.

## Supplementary Information

Supplementary Material 1.

Supplementary Material 2.

Supplementary Material 3.

Supplementary Material 4.

Supplementary Material 5.

Supplementary Material 6.

Supplementary Material 7.

Supplementary Material 8.

Supplementary Material 9.

## Data Availability

all data generated or analysed during this study are included in this published article [and its supplementary information files].

## Abbreviations

BNI: British Nursing Index
CINAHL: Cumulative Index to Nursing and Allied Health Literature
CPHIR: Consolidated Framework for Implementation Research
EMBASE: Excerpta Medica dataBASE
EMCARE: Excerpta Medica database - Nursing and Allied Health
ESAS: Edmonton Symptom Assessment Scale
ICU: Intensive Care Unit
IPOS: Integrated Palliative Care Outcome Scale
IT: Information Technology
MEDLINE: Medical Literature Analysis and Retrieval System Online
MRC: Medical Research Council
PARIHS: Promoting Action on Research Implementation in Health Services Framework
PCOM: patient-centred outcome measures
PDSA: Plan-Do-Study-Act
POS: The Palliative Care Outcome Scale
PRISMA: Preferred Reporting Items for Systematic Reviews and Meta-Analyses
PsycINFO: Psychological Abstracts Information Services
