# Supplementary material for: Implementing patient-centred outcome measures in palliative care clinical practice. An updated systematic review of facilitators and barriers
Source: BMC Palliat Care. 2026 Feb 12;25:66. doi: 10.1186/s12904-026-01997-2 (PMC12997956; doi:10.1186/s12904-026-01997-2)
Supplement: Supplementary file 1 — Supplementary Material 1. [file 12904_2026_1997_MOESM1_ESM.docx]

**Appendix 2. PRISMA-S Checklist**

| **Section/topic** | **#** | **Checklist item** | **Location(s) Reported** |
| --- | --- | --- | --- |
| **INFORMATION SOURCES AND METHODS** | | | |
| Database name | 1 | Name each individual database searched, stating the platform for each. | MEDLINE, Embase, Emcare (Ovid SP); CINAHL, PsycINFO (EBSCOhost); BNI (ProQuest); SCI-Expanded, SSCI, ESCI (Web of Science) |
| Multi-database searching | 2 | If databases were searched simultaneously on a single platform, state the name of the platform, listing all of the databases searched. | Databases searched separately on respective platforms |
| Study registries | 3 | List any study registries searched. | None reported for identifying studies |
| Online resources and browsing | 4 | Describe any online or print source purposefully searched or browsed (e.g., tables of contents, print conference proceedings, web sites), and how this was done. | Citations of original review in Web of Science, Google Scholar, Scopus; hand-searching reference lists |
| Citation searching | 5 | Indicate whether cited references or citing references were examined, and describe any methods used for locating cited/citing references (e.g., browsing reference lists, using a citation index, setting up email alerts for references citing included studies). | Cited and citing references examined via Web of Science, Google Scholar, Scopus |
| Contacts | 6 | Indicate whether additional studies or data were sought by contacting authors, experts, manufacturers, or others. | List of studies from expert advisory committee |
| Other methods | 7 | Describe any additional information sources or search methods used. | Hand-searching reference lists and citations; expert list |
| **SEARCH STRATEGIES** | | | |
| Full search strategies | 8 | Include the search strategies for each database and information source, copied and pasted exactly as run. | Search strategies provided in Appendix 1 (Extended data) |
| Limits and restrictions | 9 | Specify that no limits were used, or describe any limits or restrictions applied to a search (e.g., date or time period, language, study design) and provide justification for their use. | No limits applied (no language, date, publication status restrictions) |
| Search filters | 10 | Indicate whether published search filters were used (as originally designed or modified), and if so, cite the filter(s) used. | No published filters used |
| Prior work | 11 | Indicate when search strategies from other literature reviews were adapted or reused for a substantive part or all of the search, citing the previous review(s). | Search strategy tailored based on original review |
| Updates | 12 | Report the methods used to update the search(es) (e.g., rerunning searches, email alerts). | Single search run with subsequent email alerts |
| Dates of searches | 13 | For each search strategy, provide the date when the last search occurred. | First search: 14 November 2022. We updated it by a scoping search using our main keywords on 9 February 2025 to identify any new relevant studies |
| **PEER REVIEW** | | | |
| Peer review | 14 | Describe any search peer review process. | Search strategy peer-reviewed by information scientist |
| **MANAGING RECORDS** | | | |
| Total Records | 15 | Document the total number of records identified from each database and other information sources. | Approximately 20,000 records expected after deduplication |
| Deduplication | 16 | Describe the processes and any software used to deduplicate records from multiple database searches and other information sources. | EndNote used to deduplicate records |
|  |  |  |  |
| PRISMA-S: An Extension to the PRISMA Statement for Reporting Literature Searches in Systematic Reviews | | |  |
| Rethlefsen ML, Kirtley S, Waffenschmidt S, Ayala AP, Moher D, Page MJ, Koffel JB, PRISMA-S Group. | | |  |
| Last updated February 27, 2020. | |  |  |
