# Supplementary material for: Implementing patient-centred outcome measures in palliative care clinical practice. An updated systematic review of facilitators and barriers
Source: BMC Palliat Care. 2026 Feb 12;25:66. doi: 10.1186/s12904-026-01997-2 (PMC12997956; doi:10.1186/s12904-026-01997-2)
Supplement: Supplementary file 2 — Supplementary Material 2. [file 12904_2026_1997_MOESM2_ESM.docx]

Appendix 3. PCOMs in primary studies and reviews included

| **PRIMARY STUDIES** | | | |
| --- | --- | --- | --- |
| PCOM | | Studies | Freq |
| Edmonton Symptom Assessment Scale (ESAS); Edmonton Symptom Assessment System (Revised) Scale (ESAS-r); ESAS-r:Renal | | Bausewein et al. 2016; Beddard-Huber et al. 2015; Carli Buttenschoen et al. 2014; Diplock et al. 2019; Dobrina et al. 2018; Evans et al. 2020; Goyal et al. 2020; Harding et al. 2011; Howell et al. 2020; Hui et al. 2017a; Hui et al. 2017b; Ihler et al. 2019; Kamal et al. 2016; Kilonzo et al. 2015; Kilonzo et al. 2015; Krawczyk et al. 2019a; Krawczyk et al. 2019b; Lucey et al. 2013; Patel et al. 2022; Rauenzahn et al. 2017; Sawatzky et al. 2018; Schick-Makaroff et al. 2020; Schulman-Green et al. 2008; Schulman-Green et al. 2010; Spaner et al. 2017; Wu et al. 2022 | 26 |
| Palliative Care Outcome Scale (POS); POS (APCA, African); Palliative Care Outcome Scale - Symptoms (POS-S) (Renal); POS-Br (Brazilian Version) | | Bausewein et al. 2016; Collins et al. 2015; Dobrina et al. 2018; Downing et al. 2012; Dunckley et al. 2005; Greenhalgh et al. 2017; Guo et al. 2018; Hughes et al. 2003; Hughes et al. 2004; Martins Pereira et al. 2018; Nair et al. 2019; Reynolds et al. 2019; Rugno et al. 2016; Slater et al. 2004; Slater et al. 2005; Smith et al. 2017; Stevens et al. 2005; Tavares et al. 2017 | 18 |
| Integrated Palliative Care Outcome Scale (IPOS); IPOS-Renal; IPOS-Dem; IPOS Views on Care (IPOS VoC); Views On Care | | Bausewein et al. 2018; Bradshaw et al. 2021b; Ellis-Smith et al. 2018; Hogberg et al. 2019; Kane et al. 2017; Kane et al. 2018; Lind 2018; Pezold et al. 2019; Pinto et al. 2018; Sandham et al. 2022; Seipp et al. 2022; Viecelli et al. 2022 | 12 |
| EORTC QLQ-BM22 (Bone Metastases); EORTC QLQ-BN20 (Brain Metastases); EORTC QLQ-C15-PAL; EORTC QLQC30; EORTC QLQ-C30; EORTC QLQ-C30-PR25 Prostate Cancer; EORTC-QLC-C30 (Modified); EORTC-QLQ-C15-PAL; EORTC-QLQ-C30; EORTC-QLQ-C-30 (Item No. 30); EORTC-QLQ-H&N35; EORTC-QLQ-LC13 (Lung Cancer) | | Appleyard et al. 2021; Bausewein et al. 2016; Fabian et al. 2021; Friis et al. 2021; Graf et al. 2022; Mai et al. 2018; Mills et al. 2008; Oldenburger et al. 2020; Patel et al. 2022; Stewart et al. 2022 | 10 |
| FACT (Functional Assessment of Cancer Therapy); FACT-B (Breast); FACT-G (General); FACT-H&N (Head and Neck); FACT-L (Lung); Functional Assessment of Cancer Treatment (FACT)-Ovarian with FACT Gynecologic Oncology Group (GOG) Neurotoxity-13 | | Campbell et al. 2022; Donaldson et al. 2004; Fabian et al. 2021; Graf et al. 2022; Mai et al. 2018; Oldenburger et al. 2020 | 6 |
| VAS; Visual Analog Scale-Pain; Nonverbal Visual Analog Scale | | Fabian et al. 2021; Krulewitch et al. 2000; Lind et al. 2004; Muir et al. 2018; Oldenburger et al. 2020; Swart et al. 2022 | 6 |
| EQ-5D-5L; EuroQOL 5 Dimensions, 5 Levels (EQ-5D-5L); EQ-5D-5L; Euroqol EQ-5D-5L; EQ-5D-3L; EQ-5D VAS | | Appleyard et al. 2021; Coast et al. 2018; Schlichter et al. 2020; Stewart et al. 2022; Viecelli et al. 2022 | 5 |
| McGill Quality of Life-Revised Version; Part A of the McGill Quality of Life (QoL) Index; McGill Quality of Life Questionnaire-Revised (MQOL-R); McGill Quality of Life Questionnaire (MQOL) | | Greenhalgh et al. 2017; Kilonzo et al. 2015; Krawczyk et al. 2019a; Krawczyk et al. 2019b; Sawatzky et al. 2018 | 5 |
| Faces Pain Scale; Faces Scale; Wong-Baker FACES Pain Rating Scale; Wong-Baker FACES Scale | | Harding et al. 2007; Krulewitch et al. 2000; Lee et al. 2016; Ruder et al. 2010 | 4 |
| FICA: A Spiritual Assessment Tool | | O'Reilly et al. 2016; Ruder et al. 2010 | 4 |
| Numeric Pain Rating Scale; Numeric Rating Scale (NRS); Numerical Pain Rating Scale (NPRS) | | Lee et al. 2016; Oldenburger et al. 2020; Ruder et al. 2010; Schuler et al. 2021 | 4 |
| Canadian Health Care Evaluation Project Lite Questionnaire; Canadian Health Care Evaluation Project Questionnaire Lite (CANHELP-Lite) | | Krawczyk et al. 2019a; Krawczyk et al. 2019b; Sawatzky et al. 2018 | 3 |
| Functional Assessment of Chronic Illness Therapy (FACIT); FACIT Spiritual Well-Being Scale (FACIT-Sp) | | Mai et al. 2018; Oldenburger et al. 2020; Ruder et al. 2010 | 3 |
| Missoula-VITAS Quality of Life Index (MVQOLI) | | Donaldson et al. 2004; Hill et al. 2002; Schwartz et al. 2005 | 3 |
| Pain Map (Body Map) | | Mayahara et al. 2019; Patel et al. 2022; Ruder et al. 2010 | 3 |
| Palliative Care Problem Severity Scale (PCPSS); Palliative Care Problem Severity Scale Score; Palliative Care Problem Severity Score (PSS) | | Currow et al. 2014; Kilonzo et al. 2015; Rawlings et al. 2011 | 3 |
| Patient Health Questionnaire-9 (PHQ-9) | | Gabbard et al. 2021; Howell et al. 2020; Suri et al. 2022 | 3 |
| Symptom Assessment Score (SAS); Veteran Symptom Assessment System (VSAS) | | Currow et al. 2014; Friedman et al. 2022; Rawlings et al. 2011 | 3 |
| Brief Pain Inventory (BPI; Short and Long Forms) | | Howell et al. 2020; Oldenburger et al. 2020f | 2 |
| Canadian Problem Checklist; Problem Checklist | | Howell et al. 2020; Kotronoulas et al. 2017 | 2 |
| Generalized Anxiety Disorder 7-Item Survey (GAD-7) | | Gabbard et al. 2021; Howell et al. 2020 | 2 |
| Global Distress Index (MSAS-GDI); Memorial Symptom Assessment Scale (MSAS); Memorial Symptom Assessment Scale-Short Form (MSAS-SF) | | Bausewein et al. 2016; Swart et al. 2022 | 2 |
| Hospice Quality of Life Index (HQLI)-Revised | | Donaldson et al. 2004; Greenhalgh et al. 2017 | 2 |
| Hospital Anxiety Depression Scale | | Dobrina et al. 2018; Harding et al. 2007 | 2 |
| Ottawa Pain and Symptom Assessment Record (PSAR); Pain and Symptom Assessment Record (PSAR) | | Bourbonnais et al. 2004; Bouvette et al. 2002 | 2 |
| Pain Assessment in Advanced Dementia (PAINAD) Scale | | Muir et al. 2018; Ruder et al. 2010 | 2 |
| Pain Diary | | Lind et al. 2004; Mayahara et al. 2019 | 2 |
| Pain Thermometer; Vertically Oriented Pain Thermometer (VDS) | | Mayahara et al. 2019; Ruder et al. 2010 | 2 |
| Quality of Life Assessment and Practice Support System (QPSS) | | Krawczyk et al. 2019b; Sawatzky et al. 2018 | 2 |
| Abbey Pain Scale (APS) | | Ruder et al. 2010 | 1 |
| Activities Specific Balance Confidence scale | | Campbell et al. 2022 | 1 |
| Cancer Fatigue Scale | | Howell et al. 2020 | 1 |
| Cancer Needs Distress Inventory | | Kotronoulas et al. 2017 | 1 |
| Cancer Needs Questionnaire-Short Form | | Kotronoulas et al. 2017 | 1 |
| Cancer Rehabilitation Evaluation System-Short Form | | Kotronoulas et al. 2017 | 1 |
| Center for Epidemiological Studies-Depression Scale Short Form | | Campbell et al. 2022 | 1 |
| Cervical Cancer Concerns Questionnaire (CCCQ) | | Kotronoulas et al. 2017 | 1 |
| Client Generated Index (CGI; an Australian adaptation of the Patient Generated Index) | | Greenhalgh et al. 2017 | 1 |
| Cornell Scale for Depression in Dementia | | Krulewitch et al. 2000 | 1 |
| Dialysis Symptom Index (DSI) | | Gabbard et al. 2021 | 1 |
| Distress Thermometer | | Bausewein et al. 2016 | 1 |
| Edmonton Functional Assessment Tool (EFAT) | | Kilonzo et al. 2015 | 1 |
| Expanded Prostate Cancer Index Composite (EPIC) | | Wu et al. 2022 | 1 |
| Hospice Approach Discomfort Scale (Hurley) | | Krulewitch et al. 2000 | 1 |
| HRQOL | | Donaldson et al. 2004 | 1 |
| ICECAP-A (Investigating Choice Experiments for the Preferences of Older People-Capability Measure for Adults); ICECAP-SCM (Supportive Care Measure; EoL Capability) | | Coast et al. 2018 | 1 |
| Interactive Symptom Assessment System (ISAAC) | | Howell et al. 2020 | 1 |
| Kidney Disease Quality of Life (KDQOL-36) | | Gabbard et al. 2021 | 1 |
| M.D. Anderson Symptom Inventory Interference Scale | | Campbell et al. 2022 | 1 |
| Minimal Documentation System for Palliative Care (MIDOS) | | Krumm et al. 2014 | 1 |
| Pain Assessment Questionnaire for a Patient with Advanced Disease | | O'Reilly et al. 2016 | 1 |
| Pain Intensity Scale (PIS) | | Krulewitch et al. 2000 | 1 |
| Palliative Symptom Burden Score (PSBS) | | Fetz et al. 2018 | 1 |
| Physical/Emotional Symptom Tracking Tool | | Donaldson et al. 2004 | 1 |
| Profile of Mood States-11 | | Campbell et al. 2022 | 1 |
| Psychosocial Needs Inventory | | Kotronoulas et al. 2017 | 1 |
| Quality of Life in Life-Threatening Illness-Family Carer Version (QOLLTI-F) | | Sawatzky et al. 2018 | 1 |
| Rotterdam Symptom Checklist (RSCL) Version: Cancer Research Campaign Psychological Medicine Group | | Hardy et al. 1999 | 1 |
| Short-Form McGill Pain Questionnaire 2 (SF-MPQ-2) | | Gabbard et al. 2021 | 1 |
| Social Difficulties Inventory | | Howell et al. 2020 | 1 |
| State-Trait Anxiety-Depression Inventory (STADI) | | Mai et al. 2018 | 1 |
| Supportive Care Needs Survey-SF | | Kotronoulas et al. 2017 | 1 |
| Symptom and Falls Diary | | Campbell et al. 2022 | 1 |
| Symptom Assessment Checklist (Homsi et al.) | | O'Reilly et al. 2016 | 1 |
| Symptom Distress Scores (SDS) | | Kilonzo et al. 2015 | 1 |
| Symptom Representation Questionnaire | | Campbell et al. 2022 | 1 |
| Symptom Self-Assessment | | Donaldson et al. 2004 | 1 |
| Symptoms and Concerns Checklist (SCC) | | Greenhalgh et al. 2017 | 1 |
| The Herth Hope Index German Version (HHI-D) | | Mai et al. 2018 | 1 |
| The Schedule for the Evaluation of Individual Quality of Life Direct Weighting (SEIQoL-DW); Disease-Related SEIQoL-DW | | Greenhalgh et al. 2017 | 1 |
| **REVIEWS** | | | |
| **Pearson et al. 2007** | 24 QOL tools Assessment of QoL at End-of-Life (AQEL) Missoula-Vitas QoL Index (MVQoLI) Brief Hospice Inventory (BHI) Needs at the End-of Life Screening Tool (NEST) Care-Notebook Palliative Care Assessment (PACA) City of Hope QoL Tool (CoH) Palliative Care Outcome Scale (POS) Client Generated Index (CGI) Patient Evaluated Problem Score (PEPS) EORTC QLQ-C30 Quality of Life at the End-of-Life Measure (QUAL-E) Functional Assessment of Cancer Therapy – General (FACT-G); FACT-Pal Quality of Life Scale-Cancer 2 (QOL-CA) Functional Living Index – Cancer Scale (FLIC) Schedule for the Evaluation of Individual QoL (SEIQoL) Hospice Quality of Life Index – revised (HQLI) SEIQoL – Direct Weighting (SEIQoL-DW) Life Evaluation Questionnaire (LEQ) SF-36 Health Survey McMaster Quality of Life Scale (MQLS) Therapy Impact Questionnaire (TIQ) McGill Quality of Life Questionnaire (MQOL) WHOQOL-BREF | | |
| **Jordhoy et al. 2007** | A: Uni- or bidimensional instrument 1. Symptom assessment: Canberra Symptom Score card; Edmonton Symptom Assessment Schedule (ESAS); MD Anderson symptom inventory (MDASI); Minimal Documentation System – symptom assessment; Symptom Distress Scale (SDS); Symptom Monitor Pain Assessment: Home Hospice Patient Pain Assessment Instrument (HHPPA) 2. Global Visual Analog Scale: Global QoL uniscale 3. Satisfaction with care: Hospice Care Performance Inventory (HCPI); Quality of End-of-Life Care and Satisfaction With Treatment scale (QUEST) 4. Dying experience: Quality of Dying and Death (QODD) 5. Existential and/or psychological: Trent Hospice Audit Group (THAG), quality standard of spiritual care; Demoralization Scale; Life Evaluation Questionnaire (LEQ); The Life Closure Scale (LCS); The McCanse Readiness for Death Instrument; Schedule of Attitudes toward Hastened Death (SAHD); Subjective Well-Being Scale 6. Performance measure, PF+: Edmonton Functional Assessment Tool (EFAT; Staff); Palliative Performance Scale (PPS; Staff)  B: Multidimensional instruments 1. No PF: Palliative Care Core Standards (PCCS); Brief Hospice Inventory (BHI); Hospice Quality of Life Index (HQLI) Original); Palliative Care Assessment (PACA); Quality of Life at the End of Life (QUAL-E); The McGill Quality of Life Questionnaire (MQOL); McAdam Suffering in Terminal Illness 2. Physical Performance (PF+): Assessment of Quality of Life at the End of Life (AQEL); McMaster Quality of Life Scale (MQLS); Missoula-VITAS quality of life index (MVQOLI); Palliative Care Quality of Life Instrument (PQLI); Spitzer Quality of Life Index (SQLI); Hebrew Rehabilitation Center for Aged QL (HRCA-QL); The Therapy Impact Questionnaire (TIQ); Resident Assessment Instrument (RAI-PC); Problems and Needs in Palliative Care Questionnaire; Support Team Assessment Schedule (STAS; PF = Karnofsky Index); Palliative Care Outcome Scale (POS; PF = ECOG status); Patient evaluated problem score (PEPS; PF = WHO status) | | |
| **Stiel et al. 2012** | Quality of life/wellbeing: The EORTC QLQ-C30 (or EORTC QLQ-OES24 or EORTC QLQ-LC13); Single scales (VRS & NRS); MQOL; SF-36; HQLI; HRQoL; FACIT-F (or FACIT-Sp); Hospice Quality of Life Index (HQLI; 28Q; PRO); MVQOLI; SeiQoL; Euro Qol 5D; SF 12. Quality of care/satisfaction: STAS; FAMCARE; QODD. Physical symptoms and symptom control: ESAS; POS; Memorial Symptom Assessment Scale (MSAS); Rotterdam Symptom Checklist (RSCL); ICPs; Liverpool Care Pathway of the Dying (LCP); Brief Hospice Inventory (BHI); Palliative Care Assessment tool (PACA); Palliative Care Pain and Symptom Pocket Card (PCPSPC). Performance status/functional (dis-)ability: KPS; PPS; BI; ADL; ECOG. Psychological symptoms: HADS; MMSE; POMS; BDI; STAI. Decision-making and communication: DCS; DSAT. Place of death. Stage of disease/cause of death: Palliative Care and Severity Score; TISS. Mortality/prognosis/survival: PaP-S; CPPS; Flacker Mortality Index. Distress/wish to die: SDS; SIP; COPE scale on distress and vigor; Discomfort Scale for Dementia of the Alzheimer’s Type; DDRS; SAHD. Spirituality and personality: CRI; ABS; SCSORF; Puchalski’s FICA (Spiritual Assessment); Brief RCOPE. Disease-specific instruments: UPDRS; CAD-EOLD; PCAF-HIV; SIP-19. Clinical features Meaning in Life Needs: psychosocial needs inventory; lung cancer health care needs assessment; PNPC-sv | | |
| **Collins et al. 2015** | ePROMS 1. Tell Us (Any questionnaires; Any Device) 2. HealthHUB (ESAS; EQ-5D; Computer) 3. EPCRC-CSA (ESAS; EORTC QLQ-C30; BPI; PRIME-MD PHQ9; SGA 24; Tablet) 4. MyPAL (Smartphone) 5. CHES (EORTC QLQ-C30 Tablet) 6. ePROhub (MDASI; ISI; HADS; PHQ-9; EQ-5D-5L; DT; Smartphone runs with the app WeChat) 7. N.D. (PROMIS questions; Tablet) 8. N.D. (PCM version 2.0; Tablet) 9. N.D. (PMC 2.0; FACIT-F; FACT-L; Tablet) 10. N.D. (ESAS; PHQ-4; Tablet) 11. RELIEF (ESAS-r; DT; BPI; Any Device) 12. Ambuflex (EORTC QLQs C30 LC13; Tablet) 13. N.D. (ESAS; CAGE; Computer; The Edmonton Labeled Visual Information System (ELVIS)) | | |
| **Etkind et al. 2015** | European Organization for Research and Treatment of Cancer Quality of Life Questionnaire-Core 30 (EORTC QLQ-C30)  ESAS; POS. Others: HRQOL = health-related quality of life; FACT-G = Functional Assessment of Cancer Therapy - General; HADS = Hospital Anxiety and Depression Scale; MSAS = Memorial Symptom Assessment Scale; PACSLAC = Pain Assessment Checklist for Seniors with Limited Ability to Communicate; MOS = Medical Outcomes Study core questionnaire; SF36 = Short Form health survey 36; MVQOLI = Missoula-Vitas Quality of Life Index; HQLI 14 = Hospice Quality of Life Index 14; EORTC QLQ C30 Lung Cancer 13 | | |
| **Potts et al. 2018** | PROMS: Palliative Care Outcome Measures Organized by Biopsychosocial Model 1. Biological measures: Hundred Paisa Pain Scale (HPPS); Self-Management; VAS; Karnofsky Performance Status (KPS); APCA POS Pain Subscale; Brief Pain Inventory (BPI); PPQ-K (Patient Pain Questionnaire–Knowledge)/FPQ-K (Family Pain Questionnaire–Knowledge) 2. Psychological measures: General Household Questionnaire−12 (GHQ-12) 3. Social measures: Island Hospice Checklist; Picot Caregivers Rewards Scale (PCRS) 4. Multidimensional measures: City of Hope QOL; APCA POS; ESAS; WHOQOL-BREF; Medical Outcome Study–HIV (MOS-HIV) | | |
| **Harding et al. 2011** | Tools in Clinical Care/Audit: Karnofsky Performance Status (KPS) Scale; Palliative Performance Scale (PPS); POS; Symptom Distress Scale (SDS); Support Team Assessment Schedule (STAS); Palliative Care Assessment Tool; EORTC QLQ-C30; Measuring the Quality of Life of Seriously Ill Patients Questionnaire (QUAL-E); Barthel Index; MQOL; Unspecified VAS; EORTC QLQ-C15-PAL; Hospital Anxiety and Depression Scale (HADS); Mini-Mental State Examination; Therapy Impact Questionnaire; Memorial Symptom Assessment Scale Tools in Research: KPS; ESAS; EORTC QLQ-C30; Palliative care Outcome Scale (POS or African POS); PPS; EORTC QLQ-C15-PAL; STAS; MQOL; Functional Assessment of Cancer Therapy-General; HADS; SDS; Schedule for the Evaluation of Individual Quality of Life | | |
| **Parker et al. 2010** | Outcome measures specific to residential aged care facilities 1. Quality of Dying in Long Term care 35Q from QUAL-E (35Q); 1 additional items; (QOD-LTC; 11Q) and (QOD-LTC-C; 23Q; HCP or family member) 2. Family perception s of end of life care (FPCS; 25Q) 3. End of Life in Dementia Scales (EOLD): Satisfaction with Care at the End-of-Life (SWC-EOLD; 10Q) + Symptom Management at the End-of-Life (SM-EOLD; 9Q) + Comfort Assessment in Dying (CAD-EOLD; 14Q) 4. Palliative Outcome Scale (POS; 2V; 10Q; last 3 days) Outcome measures not specific to residential aged care facilities 1. Family Assessment of Treatment at End of Life Survey (FATE; 31Q; family perceptions of care in last month) 2. Toolkit After-Death Bereaved Family Member Interview (Toolkit Interview; 36Q) 3. Quality of Dying and Death (QODD; bereaved family members; 31Q) 4. Quality-of-Life Concerns in End of Life Questionnaire (QOLC-E; 33Q; prospectively by patient) 5. Modified Quality-of-Life Concerns in End of Life Questionnaire (mQOLC-E; 23Q) 6. Resident Assessment Instrument for Palliative Care (RAI-PC; 106Q) | | |
| **Mahmoudi et al. 2022** | PROMS: 115 different PROMs: generic HR-QoL instruments (n = 19), domain-specific instruments (n = 71), heart disease-specific instruments (n = 9), and heart transplant-specific instruments (n = 16) together with treatment adherence measures (n = 5). They cover different dimensions of HR-QoL and of immunosuppressive-drug experience, with diverse numbers of items, types of scales, and psychometric properties.  Generic and Domain-specific HR-QoL instruments MOS SF36 (Generic; 36) EQ-5D; (Generic; 5) World health organization quality of life-breF (WHOQoL-BREF) (Generic) Sickness impact profile (SIP) (Generic) Quality of life index (QLI) (Generic) 12-Item short form survey (SF-12) (Generic) Karnofsky performance scale (KPS) (Generic) Beck depression inventory (BDI) (Depression; 21) Hospital anxiety and depression scale (HADS) (Anxiety and depression; 14) Psychological general well-being (PGWB) (Psychological state; 22) Jalowiec coping scale (Coping; 60)  Heart disease-specific HR-QoL instruments Instrument name; Items (N); Method of administration Kansas City Cardiomyopathy Questionnaire (KCCQ); 23; self-administered Cardiac Depression Scale (CDS); 26; self-administered Minnesota Living with Heart Failure Questionnaire (MLHFQ); 21; self-administered Quality of Life Index–cardiac version (QLI); 36; self-administered  heart transplantation-specific PROMS Instrument name; No of domains & (items); Method of administration Assessment of Problems with the Heart Transplant Regimen; 26; self-administered Basel Assessment of Adherence to Immunosuppressive Medications scale (BAASIS); 5+VAS; self-administered interview Heart Transplant Intervention Scale; 63; self-administered Heart Transplant Symptom checklist; 92; self-administered Heart Transplant Stressor Scale (Jalowiec Stressor Scale); 81; self-administrated Immunosuppressant Therapy Adherence Scale (ITAS); 5; self-administered Immunosuppressant Therapy Barrier Scale (ITBS); 13; self-administered Medication Experience Scale for Immunosuppressants (MESI); 7; self-administered (< 10 min) Memphis Survey; 45; self-administered Modified Transplant Symptom Occurrence and Symptom Distress Scale (MTSOSD-59R); 59; self-administered Organ Transplant Symptom and Well-being Instrument (OTSWI); 8; self-administered interview Transplant Care Index (TCI); 6; self-administered Transplant Effects Questionnaire (TxEQ); 23; self-administered Transplant Symptom Frequency Questionnaire (TSFQ); 27;  self-administered Transplant Symptom Frequency & Distress Scale (TSFDS); 29; self-administered | | |
| **Voorend et al. 2021** | OMs: Geriatric assessment practices 1. Functional status (ADL/ iADL): GARS; Lawton; Katz-6; Lawton; Katz 2. Mobility: Gait speed; Hand grip strength; Short Physical Performance battery; Timed up and go; Falls; Four Test Balance Scale;  3. Cognition: Mini Mental State Examination; Clock drawing; 15- WVLT; Stroop Colour Word Test; Trail making test (A&B); Visual Association Test; Letter Digit Substitution Test; Assessment of numeracy; Mini Mental State Examination Clock drawing; Enhanced Cued Recall; Semantic Fluency Test; MOCA 4. Mood: Geriatric Depression Scale 5. Nutritional status: Subjective Global Assessment or SNAQ; Mini-Nutritional Assessment (anamnesis by dietician) 6. Comorbidity: Charlson Comorbidity Index; CIRS-G (anamnesis) 7. Quality of Life: RAND-36; EuroQol-5; EuroQol-5D; Visual Analogue Scale 8. Frailty: Fried frailty indicator; Groningen Frailty Index; Fried Frailty Index (includes 4 m walking test and Handgrip strength); Rockwood Clinical Frailty score 9. Caregiver burden: EDIZ-plus; EDIZ ([hetro]anamnesis by social worker) 10. Estimation of nephrologist: VAS: overall condition; Surprise question 11. Other: Cantril’s ladder, Pain score, Anxiety score; Illness perceptions questionnaire; Additional subjective cognition tests (by caregiver): IDDD; IQCODE; Neuro-Psychological Inventory; Outcome Prioritization Tool (treatment goals) ADL Activities of daily living; iADL Instrumental activities of daily living; GARS Groningen Activity Restriction Scale; 15-WVLT 15-Word Verbal Learning Test, immediate and delayed; SNAQ Short Nutritional Assessment Questionnaire; CIRS-G Cumulative illness rating scale for geriatrics; EDIZ ‘Ervaren Druk door Informele Zorg’ Self perceived burden from informal care; IDDD Interview of Deterioration in Daily life Dementia; IQCODE Informant Questionnaire on COgnitive DEcline. | | |
| **Eijsink et al. 2023** | Generic measure: EORTC QLQ-C30; EQ-5D; EQ-5D-5 L; CarerQoL-7D; Ferti-Qol9; EQ-6D without the cognitive dimension (transformation to EQ-5D); RAND-36; PROMIS-Global 10 Score; SF-12; SF-36 Domain-specific measure: Analogue pain scale; PHQ-9; Fatigue; HADS; CIS-20; PSS-10; BSSC-W; FSFI; FSFD-R; Individual score: Productivity, Disease control and QoL; MHQ; Modified Charlson co-morbidity index VAS Scores; Pain Intensity Scale; PDQ; TRANS-Q; MD-HAQ; VAS-pain; VAS-fatigue; PHMH; Physical function Pain interference Depression; Quality of death; RCS-NL; Vancouver Scar Scale: Patient observer Scar assessment scale; VAS Scores; WPAI fatigue Disease-specific measure: BAROS; BCTQ; BSHC BSHC-B Burn outcome questionnaire; Chronic treatments; Curative treatments; EORTC QLQ-C30; EORTC-QLQ-BR23; BREAST-Q; EORTC-QLQ-Core; HOOS-PS; KOOS-PS; Individual questionnaire: PROMs, PREMs, patient satisfaction; mHI-CD; mHI-UC; mRS; Palliative treatments; Patient satisfaction questionnaire | | |

Although measures such as the EORTC, FACIT, and EQ-5D were originally developed for research purposes rather than routine clinical use, they are included in this review due to their frequent application in pilot studies and the inclusive scope of our analysis.
