# Supplementary material for: Implementing patient-centred outcome measures in palliative care clinical practice. An updated systematic review of facilitators and barriers
Source: BMC Palliat Care. 2026 Feb 12;25:66. doi: 10.1186/s12904-026-01997-2 (PMC12997956; doi:10.1186/s12904-026-01997-2)
Supplement: Supplementary file 3 — Supplementary Material 3. [file 12904_2026_1997_MOESM3_ESM.docx]

Appendix 8. Facilitators and barriers regarding finantial costs

| **Study Name** | **Financial Costs** |
| --- | --- |
| **Appleyard et al. 2021**^30^ | - ePROs are to form a part of healthcare resource rationalisation by **replacing** some routine follow-up. - This programme makes use of physician associates to coordinate remote follow-up and **reduce** both phone and face-to-face appointments. |
| **Bausewein et al. 2016**^2^ | - Facilitator: Many of the measures developed for palliative care are **free to use**, although sometimes registration is necessary. This is important as professionals have stated that cost is a major issue when considering which tool to use. - Barrier: Instrument developers and software developers are pushing for **copyrighted** instruments, many of which then incur ongoing costs for use. |
| **Bouvette et al. 2002**^37^ | - The process to **replace** the preexisting tool with the PSAR was arranged with team leaders and/or educators, depending on the facility. - In the case of community nursing agencies and hospices that did not have a pre-existing tool, the PSAR offered a format for **recording data**. |
| **Bush et al. 2018**^39^ | - The cost was not a specific focus of most of the studies. - Approaches that involve screening records or incorporating extra technology are likely to result in increased clinical and administrative costs. The results of a recent quality improvement initiative conducted in a large academic, urban healthcare system concluded that incorporating **pay-for-performance incentives** can be used to expand PC service to the underserved efficiently, but there were substantial administrative costs. - While effective PC is associated with **overall healthcare savings**, which may be realized in the longer term, in the short term, implementation is costly. |
| **Currow et al. 2014**^45^ | - By controlling for **patients’ overall physical status** (which is the major predictor of **resource utilisation** at the end of life) in the comparisons made, residual variations are largely going to be due to variations between services: **models of care, clinical competencies, resourcing or combinations of these factors**. |
| **Donaldson et al. 2004**^50^ | - Facilitator: More **self-management**, possibly more **efficient use of resources** - Barrier: Time and **reimbursement**. In the United States, where care is frequently reimbursed by billable units of service, time spent gathering or interpreting HRQOL information as part of the clinical encounter is not built into reimbursement by third-party payers. Oncologists worry that any new activity may lengthen the patient visit by raising new patient issues, may impair their ability to see their patients in a timely way, may increase operational costs, and may reduce net income. - Facilitator: In the United States, outcomes measurement will need to be reimbursed if this is not to remain a major obstacle to its use. - Barrier: Major challenges include the availability of **resources** for **administering** the instruments and **incorporating** data into clinic workflow. - Barrier: Resources for outcomes data management and use. Ensuring that resources are available for routine data collection and management is likely to be a major challenge. An HRQOL research study may include funding for staff assistance to enlist patients, demonstrate the use of technologies, answer questions, troubleshoot technical problems, and insert results into patient records. However, institutions generally cannot add these new functions to the work of current support staff such as receptionists and nurse assistants. - In addition, **resources to respond to patient problems**, such as pain services, mental health consultation, social services, nutritional counseling, and information about community resources, might need to be added or better coordinated. - Such interventions also have **implications for the cost of care, workforce capacity, and the increased administrative complexity** that is created when various **insurance plans do or do not cover** certain services that patient reports indicate are needed. |
| **Eijsink et al. 2023**^53^ | - Facilitator: Affordability and sustainability of health care can be enhanced by **scaling up successful Value-Based Healthcare interventions** on population levels. Facilitator: **Re-designing health-care pathway**. Due to developments toward an Integrated Practice Unit, treatment **costs were reduced, and savings** made it possible to offer patients further motivational activities, such as counseling or creation of a personal diary. |
| **Evans et al. 2020**^8^ | The **value** of standardized screening with the ESAS-r:Renal may be **maximized and costs minimized** by   - Facilitator: (1) **clarifying aims** to avoid inappropriate patient expectations and provider perceptions of “failure,” - Facilitator: (2) ensuring that providers have the **training and resources** necessary to respond to high symptom scores (particularly for psychosocial symptoms) - Facilitator: (3) **screening less frequently**, such as every other month or quarterly. |
| **Gabbard et al. 2021**^59^ | - Facilitator: **ePROMs use can decrease administrative burden, avoid secondary data entry errors, and offer potential cost savings**. - Participants were compensated **$25** for their time upon the successful completion of all measures. |
| **Howell et al. 2020**^73^ | - Facilitator: **Collaboratives** have been shown to be beneficial in achieving “breakthrough” improvements in symptoms and other quality improvement initiatives. **Future large-scale pragmatic trials** are warranted to test the effects of this intervention on health outcomes and **healthcare costs** in “real-world” cancer settings. |
| **Hui et al. 2017a**^76^ | - Barrier: Electronic data capture - **upfront cost of building** a system for data entry, storage, display, integration, and protection and the financial burden of maintaining and updating the system. |
| **Kane et al. 2017**^82^ | - Facilitator: The intervention **integrated** well into the busy nurse-led clinics, **complementing existing practice without requiring additional resources**. - Facilitator: The IPOS **did not lengthen clinical reviews**. - Facilitator: This intervention integrated well into busy nurse-led CHF disease management clinics complementing existing practice, did not require additional resource use and did not impede clinical practice. |
| **Krawczyk et al. 2019b**^88^ | - Barrier: Changes are being enacted within the rise of economic priorities driving current healthcare systems, where the impetus for care is influenced by requirements for **organizational efficiency and cost containment** as much as by concerns to promote person-centred care. This framing valorizes a **“bureaucratic model” of task-based care** over therapeutic relationship building, where palliative clinicians are institutionally rewarded for adhering to economic and managerial priorities rather than patient or even clinician preferences in end-of-life care. |
| **Lind et al. 2004**^91^ | - There are neither evidence nor indications that there was any decrease in the number of contacts with hospital-based home care from the patients. However, the results indicate that patients perceive an increased quality of care. Thus, the rationale for implementing pain assessment systems in palliative care should be based on quality of care rather than reduction of visits. |
| **Mayahara et al. 2019**^99^ | - The research staff member made a home visit at 9 days post-baseline to administer the final questionnaires, retrieve the tablet computer, and give a **$10 gift card** to both the patient and informal caregiver. |
| **Muir et al. 2018**^101^ | - Facilitator: **Incentivizing Process Improvement**: Pain reporting outcomes were disseminated internally across service areas to foster friendly competition among sites, along with financial incentives for positive performance. As a result, staff became more aligned with the organization's mission for ongoing improvement in the end-outcome measure of timely pain relief. |
| **Potts et al. 2018**^111^ | - Hongoro and Dinat (2011) used a cost-accounting procedure for a district hospital in South Africa and found that cost for a home visit for patients was US$71.00, 50% less than the average cost of a patient spending a day in the hospital ($142.00). - Chellappan et al. (2011) reported similar cost savings for patients in Tamil Nadu, India. Before the intervention, all 30 patients visited hospitals for acute symptom management where 76% of patients paid between 100 to more than 200 Indian rupees (INR) (US $2.25 to $4.50, 2006 rates) per hospital visit. After training the primary caregiver with the symptom management kit, only six patients (20%) made unscheduled visits to the hospital with 96.7% of the patients spending less than 100 INR (US $2.25, 2006 rates). Eighty-three percent of caregivers were satisfied with the reduced cost of medical care as a result of using the acute symptom management kit. - Facilitator: Few studies evaluated models delivered by **community health workers or volunteers**. Given the **scarce resources in low-resource** countries, these are models that, if effective, may be able to expand the reach of palliative care services dramatically. |
| **Reynolds et al. 2019**^115^ | - Barrier: Concern about the cost of using and learning about outcome measures was also an issue in this study. **Funding** **restrictions** and the **high costs of measures** have been identified as barriers to outcome measurement. - Barrier: Organisations ought to offer resources to encourage the adoption of routine outcome measurement, but this may be a problem. A recent report found that **two-thirds of hospices have had statutory funding frozen or reduced**, which for some has resulted in service restrictions. Once again, the **unique position of a charitable organisation providing health services may be relevant when selecting an outcome measure**. |
| **Seipp et al. 2022**^126^ | - Barrier: Over the **short term**, implementation requires time and resources for training, integration into documentation systems and technical equipment, but **additional work** on data collection, administration, support and ongoing training is also necessary over the **long term**. - Faciliatator: Benze et al. found high adherence of patients with advanced cancer in the use of a **smartphone application** in the outpatient setting. More research is needed on how electronic PROM can be integrated into the outpatient palliative care setting through the use of, for example, **web-based tools**. Additional financial support is required to expand the use of PROMs in specialised outpatient palliative care. |
| **Sommerbakk et al. 2016**^130^ | - Barrier: **Funding** was mentioned in all interviews as an important factor for implementing improvements. The LMCs have limited budgets. Getting extra state funding was therefore a key facilitator for establishing the palliative care unit. Part of this funding was used for staff training. A second financial facilitator was that the local hospital agreed to pay for the most expensive medication for the patients admitted to the LMC. However, other additional costs were **not reimbursed**. Consequently, the LMC could not establish the planned ambulatory palliative care team. - Facilitator: A national facilitator for stimulating the expansion of PC in hospitals has been to include PC in the **national activity-based funding system**. - Facilitator: Other hospital wards get extra **reimbursement if they refer** patients to specialist PC services in the hospital, giving them an economic incentive to do so. According to one physician, the PCU had seen an increase in the number of referred patients after this incentive was introduced. - Barrier: The **financial implications of the** **national Coordination Reform** were mentioned as a barrier to improving PC in community services. After the reform was introduced in 2012, **patients have been discharged from specialist services earlier than before**. The idea is that specialist services should **focus on acute specialist procedures** only, while relevant follow-up care is to be provided by primary care services. However, not all municipalities have the **capacity to receive patients** in need of high-level care. Municipalities, fully aware of their shortcomings, sometimes refuse to receive their patients after they have been declared ready to be discharged from specialist services. This results in a €475 fine per day per patient, paid by the municipalities to their regional health care authorities. According to both nurses and physicians at the H-PCU, this financial incentive had also led to some municipalities accepting the return of patients in order to save themselves this expense, in spite of their lack of expertise and resources. The end result had been negative for both the patients and the staff working in the hospital PCU: The speed in which the patients were readmitted to the hospital had made the nurses call it the “revolving door effect”. Although the municipalities had accepted the patients back after the H-PCU had completed treatment, the primary care services were unable to provide adequate care and the patients were soon sent back to the hospital. However, not all municipalities allowed this “revolving door effect”. The nurses at the H-PCU also described how some municipalities would pay the fine and let the patient stay in the hospital. |
| **Stiel et al. 2012**^134^ | - Outcomes assessment related to the economy or the health care system: costs, length of stay/hospitalization, reasons for admission, quality of care/quality assurance, discharge disposition, number of admissions, funding of palliative care, and facility/size. Outcome assessment of the health care system: - A) Costs: Preferable daily patient costs (effectiveness) were taken into account besides the End-of-Life Expenditure Index (EOL-EI) in the attempt to measure the outcome of the general health care system. - B) Length of stay/hospitalization: Another domain indicating quality of palliative and hospice care is characterized by the length of patient hospital stays and the amount of end-of-life hospitalization which were found in 16 publications. - C) Reasons for admission: This outcome domain summarizes 11 different assessment methods referring to reasons for admission or referral and consultations of health care providers which were gained from national health care or health assurance agency databases and clinical records. - D) Quality of care/quality assurance: Needs assessment among health care services (4), institutional assessment of the quality of care (2), and private and confidential quality assurance (1) were reported as important features in the measurement of palliative and hospice care. - E) Discharge dispositions of patients considering the number and extent of caregivers involved in the provision of care for patients after hospital discharge were reported in six publications. - F) Number of admissions: In addition to the absolute length of hospital admissions, number of admissions of patients to hospital (4) was evaluated in a few publications. - G) Funding of pc: Once the sources of the funding of palliative care or hospice services was highlighted as an outcome measure. - H) Facility/size: Aspects of the facility environment and the size of services were examined as an issue describing quality of palliative or hospice care. |
| **van den Hurk et al. 2022**^139^ | - Barrier: **Integration in the EHR/PHR is time-consuming and costly** and must be arranged separately in each individual institution. There are several levels of technical solutions to provide feedback. A multi-step approach can be followed, from simply pushing (static) feedback to the EHR, to single sign-on integration, to sophisticated deep and dynamic integration. These structural aspects are of utmost importance for the use of the results in the consultation room and therefore for successful implementation. This should be addressed at the outset, ideally within a healthcare system. - Barrier: Development and implementation of the many **applications on ePRO-symptom monitoring** in survivorship care are costly. To our knowledge, one trial on symptom monitoring in follow-up for lung cancer showed it to be a cost-effective strategy. This means that the medical profession considers the clinical evidence of the effectiveness of these applications firm enough to continue broad implementation. In our view, however, **cost evaluation is required, and cost-effectiveness models need to include questions about the value of care**. |
| **Voorend et al. 2021**^141^ | - Supporting structures - Sufficient management support providing essential resources (e.g. **time and money**) for innovations - Barrier: In relation to time, professionals perceived **a limited budget** as a barrier, despite that some believed that nephrology-tailored routine geriatric assessment would potentially save future dialysis treatment costs. |
