# Supplementary material for: Implementing patient-centred outcome measures in palliative care clinical practice. An updated systematic review of facilitators and barriers
Source: BMC Palliat Care. 2026 Feb 12;25:66. doi: 10.1186/s12904-026-01997-2 (PMC12997956; doi:10.1186/s12904-026-01997-2)
Supplement: Supplementary file 4 — Supplementary Material 4. [file 12904_2026_1997_MOESM4_ESM.docx]

Appendix 6: Lessons learned

| **Study Name** | **Lessons (Implications, Proposal, or Suggestions)** |
| --- | --- |
| **Burner-Fritsch et al**^27^ | Workflow fit is critical: success depended on how well eIPOS aligned with existing team routines—not simply on the software; regular use builds perceived value: professionals who opened eIPOS often were more likely to recognise benefits and want continued implementation; psychosocial insights are particularly valuable: free-text answers facilitated identification of psychosocial and emotional issues usually missed in routine care; early palliative care may be a better fit: ePROMs are more feasible for less-ill patients earlier in disease trajectories; team structure determines implementation success; how teams coordinate care (shared vs. individual caseloads) determines whether PROMs can be properly integrated. |
| **Lehmann et al**^29^ | Implementing PCOMs in specialist palliative home care depends heavily on the fit between PCOMs and each team’s organisational context, including workflows, documentation systems, and communication routines. Leadership engagement, open communication, and the presence of internal facilitators proved essential for fostering coherence, motivation, and consistent use. Teams required time to develop shared understanding and consistency in assessments, particularly for psychosocial items, yet many eventually experienced improved structure in consultations, more holistic assessments, and enhanced team communication. However, not all teams continued use, largely due to perceived additional workload, non-integrated software systems, and concerns about loss of individualised care. |
| **Bennett et al. 2012**^34^ | "User’s Guide for Implementing Patient-Reported Outcomes Assessment in Clinical Practice" discusses the advantages and disadvantages of options for key design issues such as selecting questionnaires; determining the frequency with which the questionnaires are administered; determining when, where, how, and to whom the results will be presented; planning the response to patient needs identified by the PROs; and evaluating the use and benefit of PROs in a particular setting. |
| **Jordhoy et al. 2007**^80^ | 1. Physical functioning is a neglected dimension in palliative care QoL measures maybe because of availability of separate physical measure or inevitable physical decline observed among palliative care patients. 2. Physical functioning needs to be clearly defined and conceptualized, and a consensus on terminology and which aspects that are relevant for palliative care is highly warranted. 3. When, and for what purpose, self-report should be preferred over observation or testing is another question, and further testing of the palliative performance status measures is encouraged. |
| **Fetz et al. 2018**^56^ | 1. The authors recommend integrating nursing staff into the implementation process at an early stage. This integration includes offering specific training in the use of the documentation interface in addition to the possibility of providing feedback and adapting the measurement system to foster its ease-of-use. 2. Clinical experts rarely consider theoretical aspects in the development of documentation systems or measurement instruments, resulting in different measurement levels for sub-items. In the present study, it became necessary to adjust graduations of the item pain to ensure its comparability to other items of the PSBS. It was further necessary to exclude the item constipation from analyses because of its non-ordinal level of measurement. To avoid methodological challenges regarding the psychometric and clinical evaluation of patient data, the authors recommend ensuring that sub-items are measured on at least ordinal verbal rating scale with comparable intervals between characteristic values, e.g., such as the Likert scale. 3. To maintain the possibility of evaluating a proxy-based measurement system with respect to its psychometric properties, it is highly recommended to add an empirically validated instrument for data collection. When evaluating such instruments for their suitability, it is important to consider a similar outcome objective and comparable item structure. From a test-theoretical perspective, it is also important to assure continuous and comparable measurement times of the second instrument to maintain the possibility of evaluating construct validity at several times of measurement. 4. The current study yielded evidence that symptom burden is subject to frequent fluctuations in its intensity within a day. Therefore, the authors highly recommend a high-frequency measurement approach of symptom burden data. Even though this approach leads to an additional workload for nursing staff, the experience gained within this study shows that it is feasible and accepted by nurses. |
| **Guo et al. 2018**^65^ | A palliative care PROM is urgently needed for ICU setting; A pediatric palliative care PROM is needed. |
| **Bausewein et al. 2018**^31^ | Following steps to implement (I) selection of outcomes of interest; (II) selection of outcome measure(s); (III) education about the measure and use of results; (IV) selection of one coordinator/facilitator; (V) who applies the measure and its periodicity. Implementation is a time consuming and long-lasting process needing continuous attention and never seems to be completed; Implementation of outcome measurement in routine clinical care touches on principles of change management. For successful change management other factors than those relating to individual professionals may be important and that more systematic use of theories in planning and evaluating quality improvement interventions in clinical practice theories may help to explain whether change is possible; Kitson distinguishes between internal belonging to the team and external facilitators coming from outside of the team. Kitson A, Harvey G, McCormack B. Enabling the implementation of evidence based practice: a conceptual framework. Qual Health Care 1998;7:149-58. |
| **Diffin et al. 2018**^47^ | Implementation may be more successful for services that offer regular opportunities to use the intervention in practice, have sufficient levels of facilitators, stimulate more staff discussion, and encourage maintenance of positive motivation. This has informed an implementation toolkit for the CSNAT intervention. Study findings highlight the importance of assessing organizational context when implementing an evidence-based complex intervention and having a plan in place to ensure practitioners remain motivated to use the new intervention. Diffusion of Innovation Theory highlights the importance of “triability”: Innovations which users can more easily try out tend to be adopted more easily. It proposes that individuals within an organization learn within the social context of other learners. People are “not passive recipients of innovations” but rather they seek to evaluate them and communicate about them. The findings also have implications for the PARIHS framework, illustrating the importance of considering individual attitudes and motivation to use new evidence-based practice. A recent revision to PARIHS (i-PARIHS) now includes a focus on “recipients” of implementation, both individually and collectively |
| **Tavares et al. 2017**^137^ | Initial decisions taken for implementation are not final and once the protocol is put in practice, several processes will need to change across time; Need for better understanding of how institutions can join efforts to assist clinicians and services to implement PCOMs in PC clinical practice; PCOMs reflect the excellence of the health service delivered and to use them in a continuous base for quality improvement and programme certification also allows for decision and policy makers to have more concrete evidence to support and make the best decisions; When using a PCOM in PC clinical practice, individual needs, values and patient and families’ resources are taken into consideration which adds a much needed measure to help guide improvement of quality of care provided. |
| **Appleyard et al. 2021**^30^ | Introduction Stage: Introduced by trusted clinician (Stratified induction on first use depending on comfort), Appropriate timing of introduction (Provision of login details on paper and via email, User guide on paper and electronically); Usa Stage: Value reinforced by reference to results in clinic consultations (Intuitive interface), Staff familiar with system from patient perspective to troubleshoot issues (Automatic completion confirmation), Clinical resourcing to deal with issues reactively outside of clinical appointments (User-friendly presentation of longitudinal results for clinician and patient, Provision of targeted self-management resources); Widening access Stage: Prospective identification of patients less able to complete (Adjustable font size/read aloud options), Retrospective identification of non-completers and support, e.g. by phone (Provision of tablet/data allowance). If PROMs to be routine, it is imperative to find strategies to widen participation across patient groups. On-site completion is achievable in this population with limited staff support; Remote completion requires further work to improve systems and acceptability for patients. |
| **Nair et al. 2019**^102^ | Key Considerations for Successful Implementation and Uptake of PROMs Into Patient-Centered Kidney Disease Care 1. Sample characteristics of patients likely to receive greatest benefit from PROMs: Unknown to the nephrologist, clinic, or dialysis facility; Uncertain disease prognosis; Newly diagnosed with kidney disease; Recent dialysis start; Recent kidney transplant; Advancing CKD and approaching dialysis; Failing kidney transplant; Multiple comorbid conditions; History of behavioral issues; Increasingly caregiver dependent; Extremes of age 2. Method and mode of collection: Self-administered online surveys via tablet computer or smartphone; Nurse-administered online surveys via tablet computer 3. Setting and time of collection: Clinic intake room before appointment (CKD/transplant); Dialysis facility during dialysis treatment; Home during home dialysis session; At home between clinic appointments or dialysis sessions (via ecological momentary assessment) 4. Storage: Integration into electronic medical record with password-protected access; Interpretation; Incorporation into risk prediction models; Comparison with national benchmarks (ie, via US Renal Data System=USRDS data) 5. Dissemination: Regular sharing of results with patients, caregivers, and clinicians; Action; Targeted, individualized treatments based on results (referral to psychologist, change in dialysis prescription, etc) |
| **Lind et al. 2004**^91^ | There are neither evidence nor indications that there was any decrease in the number of contacts with HBHC from the patients. However, the results indicate that patients perceive an increased quality of care. Thus, the rationale for implementing pain assessment systems in palliative care should be based on quality of care rather than reduction of visits. |
| **Pinto et al. 2018**^13^ | A novel finding is that patients, family caregivers and health professionals valued the objective feedback from PCOMs, even in the face of deteriorating health. Participants agree that PCOMs support better recognition of patients’ needs and improve communication; this finding resonates with existing work. In addition, patients in our study reported feeling safe and reassured as a benefit of using PCOMs. There is an important distinction between patient burden and patients being too unwell to complete outcome measures – these are often conflated. We need more research to develop acceptable ways to interpret and use data from PCOMs with patients whose health is deteriorating. It is important to conceptualize and measure implementation outcomes to determine the effectiveness of implementation strategies. We need more quantitative research to measure these outcomes systematically. We need to recognise that patients, families and professionals may have differing views about the advantages and disadvantages of using outcome measures, particularly in relation to feelings of reassurance and burden. |
| **Radionova et al. 2020**^112^ | A significant portion of the results touch upon the symbolic acceptance of the suggested system, which stands for an increasing standardisation and technisation of medicine where interpersonal contact and the professional expertise are marginalized. All interviewees have in common that for them the planned system cannot and must not replace a personal conversation with patients. |
| **Reynolds et al. 2019**^115^ | Action research provided a structure to involve therapists in introducing outcome measures within a practice setting. This study provides insight into how a clinical speciality's discrete characteristics influence the process of selecting outcome measures. In a hospice setting, where people have life-shortening conditions, the challenges for using outcome measures are different to other settings. Palliative rehabilitation is about living with dying, rather than recovery. There continues to be a discrepancy between the mandate to occupational therapists' and physiotherapists' to use outcome measures and their practice. While the reasons for this discrepancy could be determined, addressing those issues was not so easy. Different specialities have different challenges and concerns; it is important to be aware of particular issues for a speciality when selecting and implementing outcome measures in practice. |
| **Coast et al. 2018**^42^ | Adaptive preferences may be problematic for decision makers if they rely on self-assessed measures. Although patients clearly adapted to their failing health state, for the most part their self-assessments reflected their pre-adaptation state, suggesting that these measures are appropriate for use in economic evaluations of interventions at EoL. Self-assessment of health and capability at EoL can generally be expected to give an assessment that reflects patients’ health and capability as others might see it, and researchers can continue to use this approach. |
| **Currow et al. 2014**^45^ | Bespoke measures important to patients and their families cannot be derived from clinical records and need to be collected prospectively. By controlling for patients’ overall physical status (which is the major predictor of resource utilisation at the end of life) in the comparisons made, residual variations are largely going to be due to variations between services: models of care, clinical competencies, resourcing or combinations of these factors. This has allowed a process of embedding quality systematically across a whole sector of the health system relatively quickly. Developing a culture of rapid evaluation and re-evaluation after adjusting local models of clinical care delivery is an exciting development within hospice/palliative care. Meaningful outcomes can be routinely collected in hospice/palliative care and, that by providing a feedback loop and service to service benchmarking, patient-focused improvements can be delivered. |
| **Dobrina et al. 2018**^49^ | Changing the practice is complex and requires time, continuing support, and quality in the methodologies of change enacted. All members of the team agreed to voluntarily and actively participate in the AR project; only the physiotherapists and the social worker did not participate because of their responsibilities in other units of the hospital in which the hospice was located. |
| **Friis et al. 2021**^58^ | Clinical significance of PROs are not easily interpreted. |
| **Diplock et al. 2019**^48^ | Differing cutoffs—should be incorporated into ESAS utilization, if it is to be an effective instrument to improve outcomes. This study supports the need for observing and integrating lessons learnt when implementing the use of a standardized tool like ESAS to clinical care, including seeking relevant patient and staff feedback. Need for standardized use and response to ESAS by the oncology team, to initiate symptom management conversations and determine patient referral and follow-up. While ESAS was the reasonable first choice as a broad symptom assessment tool with provincial implementation underway, future patient-centered studies should also consider symptom assessment tools with disease- and treatment-specific modules that can be appended to the general symptom list, such as the MD Anderson Symptom Inventory. LES:At the same time as introducing ESAS screening, the cancer center underwent a clinical care redesign based on the LEAN principles in ambulatory nursing. There was no actual reallocation of nurses, reduction in shifts, impact on wait times, or reduction in one-on-one time with patients with the redesigned model-of-care. There was simply a change from doctor-nurse partnership-based care to site-based care with all oncologists present in one clinic and nurses distributed across clinics to provide care as needed. |
| **Graf et al. 2022**^62^ | ePRO survey can be used even in patients with a high burden of disease as well as in older patients |
| **Martins Pereira et al. 2018**^98^ | From an ethical perspective, by either excluding patients with dementia or cognitive impairment from their study samples or by performing a proxy-assessment of needs and outcomes, it seems that some vulnerable patients are at risk of developing further vulnerabilities. According to Kipnis, there are seven categories of vulnerabilities: incapacitational or cognitive (when the person lacks the capacity to deliberate and make a decision), juridical (when the person is declared to be legally incompetent to make decisions), situational (when the person is in a situation in which medical exigency prevents the time, education and deliberation needed to make a decision), medical (when the person has a medical, serious health-related condition that may increase vulnerability), allocational (when the person lacks in subjectively important social goods), social (when the person belongs to a group whose rights and interests are socially disvalued), and deferential (when the person may be at risk of having a deferential behaviour and agrees on something regardless of his/her willingness to actually do so). |
| **Hughes et al. 2003**^74^ | Future research questions should consider the broader social, cultural and structural settings that can impact upon the use of outcome measures in palliative care. These wider contextual issues raise important issues for the development of outcome measures, including assessments of reliability and validity within different social, cultural and structural contexts between and within organizations over time.  The key message from this paper is that further research should try to map out the bigger picture of outcome measures in palliative care. The drive towards outcome measures is likely to continue and it is important that outcome measures are not developed in a vacuum, divorced from clinical practice. Rather, they should be informed and shaped by the needs and experiences of individuals and organizations involved in the delivery of palliative care. If this can be achieved then the longterm can yield outcome measures well suited to palliative care, which in turn improves care quality. |
| **Sawatzky et al. 2018**^119^ | Importance of understanding the health care organization context, including alignment with priorities and purposes regarding current and past use of QOL assessment instruments in the organization. |
| **Bookbinder et al. 1996**^35^ | Integrating a new routine into daily clinical practice takes lime and cues. Valid and reliable tools for patiens who cannot self-report pain in language that is readily understood are needed. |
| **Muir et al. 2018**^101^ | Integrating the upgraded EMR system into routine practice and workflow was critical to facilitating rapid recognition of escalating pain and inadequate pain management as well as allowing improving monitoring of patient outcomes and staff performance. |
| **Ruder et al. 2010**^116^ | It is important to remember that even when using a validated behavioral scale, the clinician should always first ask the patient for a self-report of pain. Goal of pain management at end of life, whether the pain is caused by cancer or by other causes, is comfort. |
| **Ellis-Smith et al. 2018**^54^ | Key mechanisms of action were: improved observation and awareness of residents, collaborative assessment, comprehensive ‘picture of the person’, systematic record keeping, improved review and monitoring, care planning and changes to care provision, and facilitated multi-agency communication. Potential benefit included improved symptom management, improved comprehensive care, and increased family empowerment and engagement. Apart from at the baseline and final time points, the research team was not involved and did not prompt care home staffs’ use of IPOS-Dem through the course of the implementation phase. This was to understand the implementation of IPOS-Dem without the use of facilitation which is frequently not available or sustainable in under-resourced care settings.  Few professional participants expressed concern regarding feasibility with some considering the potential usefulness of using IPOS-Dem as frequently as weekly. Family participants, however, worried that care home staff may not have the motivation or time to use IPOS-Dem, and the potential for it detracting from their caring responsibilities. Our study also gained an understanding of potential risk of harm of using a measure in routine care. BAR: Participants did identify risks of inaccurate assessment either due to poor assessment or lack of measurement reliability, corroborating a finding of a previous study. |
| **Potts et al. 2018**^111^ | Many palliative care intervention models exist to serve patients in low-resource countries. Yet, limited high-quality evidence from low-resource countries is available to document intervention outcomes. The gap identified through this review was not the lack of models for palliative care in low-resource countries, but the lack of evidence from rigorously designed experimental studies. |
| **Hawley et al. 2011**^70^ | Modifications to the scale have been made to improve clarity and allow for the expected drop in bowel activity seen in end-of-life care. After recruitment to the study, one of the four outpatient clinics was unable to implement the BPS because of staffing changes and so was dropped from the study. One of the recruited hospices was unexpectedly closed down, but another hospice in the same regional health authority was recruited in its place. Scale was easy to implement in the outpatient setting, but was more challenging in the inpatient sites, and required significant nurse education. |
| **Collins et al. 2015**^43^ | Most e-PROMS were administered through a touchscreen tablet and a pen/stylus. ePROMS - The acceptability and feasibility of electronic PRO assessments are influenced by patients’ physical condition. Several factors may influence the success of the implementation of electronic PRO data assessment in oncology palliative clinical practice, such as cultural and socioeconomic factors and the e-health literacy and care setting (inpatient vs. outpatient), because patients’ goals and care needs often differ. It would be interesting to involve palliative cancer patients at home in studies testing electronic PRO data assessments and even compare the populations of inpatients and outpatients considering the different settings. |
| **Kamal et al. 2016**^81^ | Movements toward a rapid learning health system leverages other data inputs like patient-reported data to complement clinician-entered data and provide a more comprehensive view of the quality of care delivered. |
| **Evans et al. 2020**^8^ | Our findings on patient-provider communication are consistent with a review that found that PROMs did not shift providers’ communication practices but did positively influence patient-provider communication in other ways, such as empowering patients to raise issues with providers. |
| **Bush et al. 2018**^39^ | Patient-reported data, including current symptoms, were incorporated in the EHR and could be used by providers for clinical symptom management and EOL decision making. All studies recommended additional refinement of algorithms and workflows. Feedback from patients, caregivers, and healthcare providers highlight the need to enhance interoperability among disciplines. The technology is available in the EHR, the EHR is currently underutilized for PC CDS, facilitating patient-reported outcomes, and capturing ACP. The review indicates despite the increased focus placed on using the EHR to identify PC patients more rapidly, and to incorporate patient wishes and reported outcomes in the EHR, there is the need for greater inclusion. The patients studied were largely unrepresentative of general populations. Studies in the US were largely in academic medical systems and participants also tended to be white, have health insurance, and where reported, to be of higher education and income levels. Very few studies in predominantly English-speaking countries reported having any PC materials in languages other than English. More research is needed examining the associated low completion rates, feedback regarding patient-facing technology, and clinical value. Further studies of the role of CDS and PROMs to identify appropriate patients, establish care goals earlier in their illness as well as the potential to reduce provider discomfort when introducing the topics of PC, ACP, death, and dying are needed. Earlier and more effective PC identification can also help providers, patients, and families to discuss EOL options to match with the best type of care according to patient goals and EOL stage, improving comfort care and allowing provider to focus on offering the best intervention. |
| **Lind et al. 2008**^92^ | Patients perceived an increased and improved contact with their caregivers. It further expresses how the patients took a greater part in their own care, and that they had a sense of increased security. They linked the increased and improved contact with their caregivers, as well as their increased participation in the care, to a sense of increased security. The belief that the system and transferred assessments were monitored by the caregivers around the clock also contributed to the patients’ feelings of increased security. |
| **Garcia et al. 2019**^60^ | Patients who triggered clinical alerts tended to be younger and more recently diagnosed, to have greater comorbidities, and to be a racial/ethnic minority (Hispanic, African American/black, or other race). Patients who triggered clinical alerts had more health care service encounters in the ensuing month. |
| **Oldenburger et al. 2020**^104^ | PROMs were exclusively used as a benchmarking tool and never to improve symptom control or QoL for individual patients (Palliative RT). The great variety in the way PROMs are used in palliative RT. However, we also discovered that PROMs were exclusively used as an endpoint in a study and are not used in routine care. PROMs were always used for follow-up or benchmarking, but never to improve symptom control or QoL for an individual patient. Additionally we saw that ePROMs were not used in this population. A clear guideline on the use of PROMs in the palliative RT setting may be impossible, due to the large variety in pathologies, radiation schemes and treatment indications. This, however, does not excuse researchers not to make PROMs a priority while writing a study protocol. |
| **Greenhalgh et al. 2017**^63^ | Providers with more experience were more likely to make sustainable improvements (in QI context). When change did occur, efforts were more likely to be directed at less complex, discrete organisational aspects of care. Changes that required clinicians to modify the interpersonal aspects of their care, or that were more complex, were perceived as more difficult to implement. Changes to one aspect of patient care could have unintended effects on other aspects of care. Significant and sustained improvements in patient care in response to the feedback of performance data can be achieved only through system- and organisation-wide strategies. Whether PROMs support or constrain patients in sharing or raising issues with clinicians depends on the structure of the PROM. Standardised PROMs were useful for those patients who preferred not to talk about personal or sensitive issues, helping them to share information.  Clinicians and patients perceived that, when used in first assessments, individualised PROMs supported relationship-building because they enabled the patient to ‘tell their story’. PROMs feedback could increase discussion of symptoms during the consultation and, in one study, led directly to improvements in patient well-being. The mechanism underlying this process is that patients felt more comfortable raising both physical and psychosocial or non-medical issues with their doctors and were more likely to initiate discussion about these during the consultation. Patient-reported outcome measures do not substantially change doctors’ communication practices during the consultation. Consultations still focused on symptoms rather than psychosocial issues, and clinicians were not more likely to initiate discussions about the latter. This was because doctors see their remit as dealing with issues specifically related to the patient’s condition and its treatment, and consider that it is nurses’ role to address wider psychosocial issues, a perception shared by nurses themselves. The limited effect on discussion also occurred because doctors closed down discussions about issues they felt unable to treat. In palliative care settings, although nurses recognised that PROMs could raise issues that fell outside their remit and that they could not address, they recognised that ‘just discussing’ these issues could have therapeutic value for patients. There is also tension between PROMs as a QI strategy versus their use in the care of individual patients; PROMs that clinicians find useful in assessing patients, such as individualised measures, are not useful as indicators of service quality. nurses perceived the PROM to be ‘a positive complement but not an alternative to other nursing assessments. |
| **Schulman-Green et al. 2010**^123^ | Recommendations to promote effective use of ESAS data for quality monitoring of hospice care include standardizing implementation procedures, adding patients' preferences to the ESAS form, and staff education to enhance comfort with the instrument before implementation. As ESAS data may be considered for benchmarking of hospices nationally, consistency in implementation procedures is paramount. A certain amount of standardization in quality assessment is necessary and desirable; however, in the hospice setting, the need for standardization must be balanced with the priority of individualized care. |
| **van den Hurk et al. 2022**^139^ | Regardless of the infrastructure that is used for ePRO collection, international developments can be unlocked by providing controlled access to ePRO data, algorithms, and models. The use of ePROs is instrumental in driving innovation in the eHealth era. For symptom management, it is shifting care from reactive to proactive and preventive by integrating predictive devices into the patient’s daily life. |
| **Donaldson et al. 2004**^50^ | Removing one constraint may not be sufficient, and progress will surely require multi-level interventions that address the clinician-patient visit, the clinical micro-system, and the larger health care organization in which it may be embedded. HRQOL can be measured, but that it should be measured by nonphysicians.  They underscored the need for implementation strategies that used well-grounded approaches to individual and organizational change. The purpose of assessment may affect the choice of instrument, patient population, timing, and frequency of administration. If implementing complex systems all at once is not realistic, it may be possible to devise a “good enough” strategy and implement it on a small scale, evaluate its strengths and weaknesses, and increase its scope as warranted. As clinical leaders in some organizations devise successful structures, they could share their experience professionally and provide a “ toolkit” of useful approaches. The major challenge to outcome measurement may lie less in opposition to the idea of measuring outcomes than in doubt or uncertainty about how to integrate such measurement into the health care process. Two health care delivery issues will require particular attention to achieve the potential for patient-reported outcomes: information infrastructure and technologies to support it, and redesigned systems of care that focus on patients' needs and preferences. |
| **Eijsink et al. 2023**^53^ | Results illustrate the disconnection between the development of PROMs and PREMs and the implementation phase, with implementation of value-based health-care (VBHC) still in infancy. This study shows the difference in patient-reported outcomes between curative, chronic, and palliative treatments in the early development from concept to practice in Western countries. VBHC is a continuous implementation process Patient-reported outcome measures (PROMs) and patient-reported experience measures (PREMs) are used to actively involve patients in individualized therapy during the entire health-care pathway. |
| **Campbell et al. 2022**^40^ | Some study participants may derive a sense of symptom controllability through daily symptom monitoring or may find it useful for framing their symptom management discussions with the clinical team. Daily symptom and function monitoring in research studies may provide patients with information to support symptom discussions with the clinical team. |
| **Howell et al. 2020**^73^ | Strategies that are necessary to ensure practice innovations to embed PROs in “everyday” practice for patient management are sustained is an important focus for future research as we did not measure the long-term impact of iPEHOC or if uptake was maintained. |
| **Slater et al. 2004**^127^ | The completion of POS at start of the day appeared to help the patient relinquish his/her sense of responsibility over this sometimes difficult dilemma. Patients thought the staff, on receipt of the forms, became responsible for deciding the priorities of the day. |
| **Sandham et al. 2022**^118^ | The increased reliability when family supported the assessment could be due to the New Zealand context, where whānau (extended families or community of related families) are encouraged to actively participate in caring for dying relatives. Increased reliability in scoring may relate to patients being encouraged by whānau to express their experiences and feelings in more detail than through self-reflection or staff assistance. |
| **Dunckley et al. 2005**^128^ | The initial phase of implementation is the most time consuming and once measures are integrated into routine care their use may not add an unduly large burden on workloads so long as the measure itself is easy to administer. |
| **Slater et al. 2005**^128^ | The initial phase of the implementation may be the most time consuming until the practice development (i.e. outcome measure) has been integrated into routine daily practice (Dunkley et al, 2005). |
| **Kane et al. 2018**^83^ | The intervention impacted on processes of care by enabling a shared understanding of patients’ symptoms and concerns, facilitating patient–nurse communication by focusing on these unmet needs and empowering patients to become more involved in clinical discussions. PROMs function more as a tool which enables patients to raise issues rather than changing the nature of patient–healthcare professional communication. |
| **Fabian et al. 2021**^55^ | The low rate of patient-reported outcomes and standard of their use in studies of palliative radiotherapy for head and neck cancer is in part also reflected in other entities. This might be rooted in a long-recognized difficulty in defining the right outcome measure for palliation. The rate of missing patient-reported outcome data can be lower than average in head and neck cancer patients undergoing palliative radiotherapy. In our view, the use of patient-reported outcome measures should therefore not be discouraged in this setting. |
| **Collins et al. 2015**^43^ | The POS and STAS are now used in a wide variety of settings and countries. These tools may be used in the future to compare palliative care needs and quality of care across diverse contexts and patient groups. Use and translation of these tools has steadily increased. There appears to be greater popularity for the POS than for the STAS, with more than four times as many publications for the POS being published over the last four years. Having completed patient and proxy ratings available is valuable, as it enables direct comparison of perceived severity of symptoms from patient and proxy perspectives. Future psychometric studies should include testing of interrater reliability, particularly between patient- and staff-completed versions. Data on patient versus proxy ratings support the validity of measures and ensure the whole of the palliative care population can be included. |
| **Rugno et al. 2016**^117^ | The POS may be considered as the gold standard tool in the context of PC. |
| **Daveson et al. 2012**^46^ | The range of outcome domains and perspectives involved in palliative care indicates a need for interdisciplinary research teams to define important measures and approaches. Standardization and agreement of a core set of existing tools in end-of-life cancer care based on scientific rigorous criteria is needed, and that this must be balanced with diversity and flexibility. Call for a definition of important measures and approaches, and the establishment a core set of existing tools for palliative care based on scientific rigorous criteria. |
| **Stiel et al. 2012**^134^ | There is a dominance of symptom-related instruments in the assessment of palliative care outcome. The wide scope of existing instruments makes identification on a core set of instruments for outcome assessment in palliative care difficult. Consensus from a large body of international experts would be needed to overcome this obstacle and define a core set of appropriate instruments. A framework with a set of appropriate instruments could help (1) to harmonize the variety of tools used in research and clinical practice, (2) to allow for more comparability, and (3) to define gaps were tools maybe missing and should be developed. |
| **Harding et al. 2011**^68^ | There is an urgent need to rationalize core tools to those that have been developed and validated within our population and have sound psychometric properties. |
| **Etkind et al. 2015**^7^ | There was strong evidence for an impact of PCOMs feedback on processes of care including better symptom recognition, more discussion of quality of life, and increased referrals based on PCOMs reporting. There was evidence of improved emotional and psychological patient outcomes but no effect on overall quality of life or symptom burden. A key factor in the implementation of PCOMs into routine practice is the way in which PCOMs information is used. An important consideration is that this high-quality evidence related to PCOMs measurement in palliative care populations is predominantly from oncology patients in outpatient settings. |
| **Sommerbakk et al. 2016**^130^ | This analysis relies heavily on Grol and Wensing’s model of “barriers to and incentives for change at different levels of healthcare” [49]. According to Dierckx de Casterlé, “using a preconceived framework runs the risk of prematurely excluding alternative ways of organizing the data that may be more illuminating”. |
| **Hogberg et al. 2019**^72^ | Use of IPOS as a basis for conversation promotes safe care by making the patients feel confident that the care provided was adapted to them which gives them a sense of safety and being of help to others. IPOS facilitated discussions between patients and nurses about care needs. The patients believed that using IPOS enabled reflection on their well-being and life situation. |
| **Ihler et al. 2019**^78^ | When ESAS is routinely used to map symptoms, the patients experience greater symptom relief and a better quality of life. However, not everyone uses the tool systematically. |
| **Hill et al. 2002**^71^ | When revelation was acceptable to the patient, this process was often continued by analysing or sharing the reasons for their QOL responses with either the researcher or their nurse. Revealing such deep and intimate information helped to quickly create a caring, therapeutic relationship. An increasing awareness of deficits in their practice skills, knowledge and understanding, encouraged them to seek new knowledge, including hope-fostering strategies. As with pain, QOL is what the patient says it is, and exists when the patient says it does. The study suggests that nurses can further their skills in assessing patients’ QOL and achieving appropriate responses. This may be especially important when patients are too ill to communicate such information directly. |
| **Kotronoulas et al. 2017**^86^ | Women with cervical cancer perceive important benefits from participating in PROM-driven, time-protected, and private sessions with their cancer nurse specialists. |
| **Bourbonnais et al. 2004**^36^ | Much of the success related to sustaining the use of the tool came from the champions who were on the working group. When these members of the working group were given the opportunity and power in the setting to be involved in the continuing education and support of the tool, staff appeared to buy into the new tool as well. By working with these champions, staff can practice using the tool, have their questions answered and clarify how the tool can contribute to patient care. The importance of this role was underestimated and in future would be more formalized. |
| **Mills et al. 2008**^100^ | Patients may need more time to become confident in using their diary and over time they may become prepared to share it with health professionals, with privacy becoming less of an issue as their confidence in the health care staff increased. Alternatively as their condition continued to deteriorate they decided to share the diary with the health care staff as a means of seeking help and support. |
| **Bausewein et al. 2016**^2^ | Should ideally cover several domains including physical (e.g. physical symptoms and functional status), psychological (e.g. cognition and emotions), social and cultural (e.g. family and friends, organisational and financial) and spiritual (e.g. beliefs, meaning and religion) domains. Multidimensional measures – such as the ESAS or POS; Compared to patient’s self-reports, clinicians under-estimate and spouses or partners over-estimate the severity of some symptoms. more agreement concerning the more overt symptoms whereas agreement is poorer for more latent aspects of the patient’s experience. Use measures that are suited to the clinical task being delivered and also suited to the aims of your clinical work and the population you work with. They differ in the domains and dimensions they measure, and in their length, measurement window, accessibility and cost. Outcome measurement implementation in organisations can be further aided through understanding the acceptance or reluctance to use outcome measures across healthcare professions. For the clinical setting, multiple symptoms should be included in the measure in order to assess the full experience and symptom burden of the patient. One key reason for asking about a number of symptoms is that there are symptoms that patients are less likely to volunteer, unless specifically asked. Appropriateness and acceptability are used to indicate whether a measure is suitable for its intended use. There needs to be a balance between sound psychometrics and the feasibility of a measure for clinical use, otherwise referred to as the measure’s clinimetric properties. Responsiveness to change refers to whether the measure can detect clinically important changes over time that are related to the course of the disease or to an intervention, such as symptom management. The interpretability of an outcome measure refers to whether the results (which are often a number) can be translated into something more meaningful to the patient/family or clinician. The complexity of the patient group under consideration must be taken into account especially if the report is to be used for benchmarking of services. |
| **Bausewein et al. 2011a**^32^ | The need to provide interactive content with clinical cases and training examples in addition to detailed information about specific PROMs and related references. Professionals wish to communicate with each other using comments, fora, and sharing content (i.e. advice, syntax for analysis); development of competency-based training programs which are learner- or participant-centered taking into account the individual situation and the clinical environment of the professional; Mobilising national and international palliative care and professional organisations to optimise the outcomes of the delivery of eol care ensuring that this service is both delivered and measured. People would prefer simple tools relevant to clinical practice; the service-users' perspective needs involvment in tools; Respondents favoured tools in an electronic format with the possibilities to choose from different modules according to patient situation and condition; improvement of existing measures rather than the development of new tools; comparative information on different tools to ease choice; contact details and requirements of use (e.g. payment); link to validation paper and other publications; "Tools are only as good as the people who use them" |
| **Lind 2018**^94^ | The process evaluation of the implementation showed a large variation of completed IPOS, indicating a strong influence of contextual factors. Implementation in healthcare is most often a complex undertaking because it includes several interacting components. Each component may have the possibility to influence the outcome of the implementation. According to the Medical Research Council framework on complex intervention, an intervention may be defined as complex based on 5 different aspects, each one contributing to the complexity: The number of interacting components, the number of behaviour changes that the receiver/participant in the intervention are expected to undertake contributes to the complexity, as well as if the expected change is experienced as difficult, the number of groups and if the groups are at different levels in healthcare organisation also make a contribution, the number of outcomes and opportunities for tailoring intervention to context influence the complexity. |
| **Karamanidou et al. 2020**^84^ | The review of the corpus revealed (1) an archetype of ePRO-enabled interventions for palliative cancer care, which most commonly use ePROs as study end point assessment instruments rather than integral intervention components; (2) the fact that the literature has not fully embraced the modern definitions that expand the scope of palliative care; (3) the striking shortage of promising ubiquitous computing devices (eg, smart activity trackers); and (4) emerging evidence about the benefits of narrowing down the target cancer population, especially when combined with modern patient-centered intervention design methodologies. The main goals of the digital health interventions: 1. To increase the outcome reporting frequency, to provide more insights into health care rofessionals 2. To promote the self-management of patient symptoms 3. To deliver personalized treatment 4. To improve the behavior and skills of health care professionals For future digital health approaches in palliative cancer care, a good understanding of the particular needs of the target population is paramount. It should be highlighted that none of the reviewed interventions exploited modern user interfaces empowered by computational intelligence, such as conversational agents, whereas only one of them adopted a gamification approach. The reviewed studies have not taken advantage of recent advances in wearables (ie, smart watches or smart wristbands), which could nicely complement ePROs. |
