# Supplementary material for: Implementing patient-centred outcome measures in palliative care clinical practice. An updated systematic review of facilitators and barriers
Source: BMC Palliat Care. 2026 Feb 12;25:66. doi: 10.1186/s12904-026-01997-2 (PMC12997956; doi:10.1186/s12904-026-01997-2)
Supplement: Supplementary file 5 — Supplementary Material 5. [file 12904_2026_1997_MOESM5_ESM.docx]

Appendix 4: Categories and sub-categories of facilitators

| CATEGORIES | SUB-CATEGORIES | QUOTES |
| --- | --- | --- |
| Planning, process and logistics |  | “Pre-implementation planning to create an implementation strategy tailored to meet needs of individual services.”^65^ |
|  | Healthcare systems and politics | “Consistency of the implementation components with proposed recommendations from national guidelines.”^30^ |
|  | Training, knowledge, perception, and acceptance | “There is a significant need for supporting and educating clinicians to enhance their competence in introducing the use of QOL assessment instruments to patients and family caregivers reviewing QOL assessment data with them, and using QOL assessment data to inform person-centered care planning and shared decision making.”^119^ |
|  | Organisational culture and structure | “Team discussions about the value of measurement may aid implementation and the facilitation of an organisational culture that is committed to measurement.”^2^ |
|  | Team and champions      Accountability      Leadership | “Formally appointed implementation leaders (champions, coordinator); use of internal and external facilitators with formal assignments.”^49^  “Involving staff in implementation and facilitating individual responsibility, ownership and motivations to use PCOMs.”^126^  “senior staff and managers, actively participating in the implementation and use of PCOMs.”^13^ |
|  | Personal characteristics | ““Personal beliefs and clinical experience were key aspects shaping the views of health professionals regarding the use of PROMs in palliative care.”^43^ |
|  | Patient factors    Patients’ safety | “...palliative care patients may adopt either a tolerating or an adapting coping style and that this may influence their engagement with PROMs completion: Patients using an adapting style may feel more able to confront their problems, while those using a tolerating style may prefer the opportunity to escape from their sick role.”^63^  “Remote patient has internet connection to pass on the assessment to the nurse in case an immediate action is required”^84^ |
|  | Communication | “structured reflection meetings were used as a mechanism for teams to discuss implementation progress and identify solutions collaboratively”^38^ |
|  | Information flow, action, visible outcomes | “Findings from continuous measurement are applied to service improvement and quality assessment.”^65^ |
|  | Time needed to implement | “Allocation of longer time for implementation in line with the principles of adult education and continuous use.”^38^ |
| Resources |  | “The presence of supportive infrastructure, organizational readiness and clear implementation strategies were associated with the success of ePROMs interventions.”^84^ |
|  | Time | “...shorter time spent completing the ePRO assessment than the paper version.”^44^ |
|  | Costs | “By integrating PROMs into the EHR and automating their administration and scoring, we can enhance efficiency, reduce documentation burden, and improve clinical decision-making. There is also potential for long-term cost savings by identifying issues early and tailoring care more precisely.”^57^ |
|  | Staff | “Achieving staffing levels appropriate to the level and diversity of patients’ demands.”^84^ |
| PCOM |  | “...completing the PROM gave patients confidence that their feelings mattered; patients may be empowered by the use of the instrument, since it makes it easier for the patients to voice their concerns.”^63^ |
|  | Timing for measurements | “Being flexible in the timing of administration to allow for fluctuation in a person's ability to perform activities near end-of-life, and speedily administered measures, enabled clinical utility.”^45^ |
|  | Equipment  Devices      Use experience and user interface | “Kiosks and tablets were implemented to allow for real-time reporting of symptoms directly to clinicians.  Mobile phone technology could revolutionise the collection and sharing of this information.”^62^  “Desirable features focused on hardware (lightweight, durable, and easy to disinfect), software (simple, user-friendly interface, multi-linguistic, integration with e-health systems).”^119^ |
| Data related |  |  |
|  | Legality, privacy, security, and confidentiality | “Patients should be told about the use, storage, and confidentiality of their patient-identifiable information.”^84^ |
|  | Bias control | “Ensure that the access to ePRO data is not biased and does not accentuate disparities.”^139^ |
