# Supplementary material for: Implementing patient-centred outcome measures in palliative care clinical practice. An updated systematic review of facilitators and barriers
Source: BMC Palliat Care. 2026 Feb 12;25:66. doi: 10.1186/s12904-026-01997-2 (PMC12997956; doi:10.1186/s12904-026-01997-2)
Supplement: Supplementary file 6 — Supplementary Material 6. [file 12904_2026_1997_MOESM6_ESM.docx]

Appendix 5: Categories and sub-categories of barriers

| CATEGORIES | SUB-CATEGORIES | QUOTES |
| --- | --- | --- |
| Healthcare professionals | Attitudinal | “…they may have less opportunity to convene team members for regular discussion of how to overcome any barriers to implementation, which may lead to attitudes to the intervention becoming more negative over time.”^47^ |
|  | Fatigue | “Outcome measures were introduced step by step in the participating organisations, and this may have led to a more protracted adoption process or implementation fatigue; influencing professional views.”^13^ |
|  | Training  High workload  Reluctance to use  Perceived interference | "Many participants admitted not fully understanding the ESAS tool, indicating a need for better training and education."^41^  "Staff were concerned about the increased workload associated with collecting and acting on outcome data, particularly within already time-pressured environments."^38^  "There was uncertainty among professionals about how outcome measures could actually benefit practice, leading to low motivation to use them."^74^  "Some clinicians viewed outcome measurement as a bureaucratic intrusion into their clinical autonomy and professional judgement."^130^ |
| Patients | Too unwell | “Some patients were too ill to complete the questionnaires, which made data collection inconsistent and added burden to clinicians."^32^  “Patients nearing the end of life may find it challenging to relate to abstract quality of life concepts embedded in outcome measures”^42^  “Patients often experience difficulty understanding the scales or may not be willing to report symptoms honestly."^41^ |
|  | Complexity of disease trajectory  Need for clarity  Lack of reminders |  |
|  |  |  |
|  |  |  |
|  |  |  |
| Resources | Time constraints | “… participants spent time deliberating over, as opposed to efficiently using, the measures.”^38^ |
|  | Costs | “The cost of implementation was also a concern, particularly when considering integration with the electronic health record and the resources needed for training and support.”^60^ |
|  | Equipment  Access to tool  Sustainability issues | “…failure of electronic devices (e.g. dead tablet batteries), lack of space for the device on mobile treatment carts, and device software issues.”^73^  “…“...staff in the care homes did not have access to the original version of the IPOS-Dem guidance document…”^54^  “Challenges to sustainability included lack of funding and inadequate staffing”^54^ |
| PCOMs | Outcomes complexity  Measure complexity, namely issues with adaptability; interpretation; individualisation; comprehensibility; timing; numerical scoring; user experience; acceptability; consensus on what tool to use; does not reflect reality; psychometric properties; burden | |
|  |  |  |
|  |  |  |
| Health service | Organisational culture, regarding difficulties with leadership, accountability, relationship complexity, information flow, lack of champions, ethics, data-related issues, uncertainties, privacy and confidentiality | |
| Healthcare system | Legal issues  PC issues | |
