# Supplementary material for: Implementing patient-centred outcome measures in palliative care clinical practice. An updated systematic review of facilitators and barriers
Source: BMC Palliat Care. 2026 Feb 12;25:66. doi: 10.1186/s12904-026-01997-2 (PMC12997956; doi:10.1186/s12904-026-01997-2)
Supplement: Supplementary file 7 — Supplementary Material 7. [file 12904_2026_1997_MOESM7_ESM.pdf]

Appendix 1. Search strategy for MEDLINE

Ovid MEDLINE(R) and Epub Ahead of Print, In-Process, In-Data-Review & Other Non-Indexed Citations, Daily and Versions <1946 to November 08, 2022>

1 exp Terminal Care/ or exp Palliative Care/ or exp "Hospice and Palliative Care Nursing"/ or exp death/ or exp palliative medicine/ or exp terminally ill/ or exp hospice care/ 265966

2 ((end adj2 life) or ((final* or last*) adj1 (hour* or day* or minute* or week* or month* or moment*)) or palliat* or terminal* or (end adj stage) or dying or (body adj2 (shutdown or shut* down or deteriorat*)) or deathbed or ((incurable or irreversibl*) adj2 ill*) or advanced stage or hospice*).ti,ab,kw,kf. 767171

3 1 or 2 960375

4 (PROS or PROMS or patient reported outcome* or self-report* measure* or patient report* outcome* or patient centred outcome* or patient centered outcome* or (outcome* adj3 (measure* or scale* or score* or assess* or preference* or project*))).ti,ab,kw,kf. or standards.fs. 1203224

5 (apca or "Outcome Scale" or campas-p or ("comprehensive measure" adj4 professionals) or campas-r or ("comprehensive measure" adj4 patient*) or coop or "Dartmouth Primary Care Cooperative Information Functional Health Assessment" or ecogs or "Eastern Cooperative Oncology Group scale" or eortc or "European Organization for Research and Treatment of Cancer" or eq-5d or "European Quality of Life-5 Dimensions" or esas or "Edmonton Symptom Assessment Scale" or fact-l or "Functional Assessment of Cancer Therapy-Lung" or ipos or (integrated adj4 "outcome scale") or kps or "Karnofsky Performance Score" or mcpac or "multidimensional continuous pain assessment chart" or mvqoli-r or "Missoula VITAS Quality of Life Index-Revised" or msas or "Memorial Symptom Assessment Scale" or nrs or "numeric rating scale" or paca or pcpss or "Problem Severity Score" or pcoc or "Outcomes Collaboration" or pos or " Outcome Scale" or psar or "Ottawa pain and symptom assessment record" or "psq c" or "Patient Satisfaction Questionnaire C" or qlq-c30 or rcsl or "Rotterdam symptom checklist" or rug-adl or "Resource Utilization Group Activities of Daily Living" or sf-36 or "Medical Outcomes Study 36-Item Short-Form Health Survey" or vas or "visual analogue scale" or wonca or "World Organization Project of National Colleagues and Academics" or (pain adj (chart or scale))).ti,ab,kw,kf. 154412

6 4 or 5 1325308

7 exp Implementation Science/ 1168

8 ("use" or utilis* or utiliz* or useful or feasib* or integrat* or translat* or complian* or adapt* or challeng* or impact* or captur* or transfer* or feedback* or agree* or (lesson* adj1 learn*)).ti. or (implement* or constrain* or tool* or collaborat* or cooperat*).ti,ab,kw,kf. 3461650

9 7 or 8 3461691

10 3 and 6 and 9 7832

11 exp animals/ not (exp humans/ and exp animals/) 5062632

12 10 not 11 7703
