# Supplementary material for: Implementing patient-centred outcome measures in palliative care clinical practice. An updated systematic review of facilitators and barriers
Source: BMC Palliat Care. 2026 Feb 12;25:66. doi: 10.1186/s12904-026-01997-2 (PMC12997956; doi:10.1186/s12904-026-01997-2)
Supplement: Supplementary file 8 — Supplementary Material 8. [file 12904_2026_1997_MOESM8_ESM.docx]

# Appendix 7. Frameworks, Models, and Theories Used to Support PCOM Implementation

This appendix presents the implementation frameworks, models, and theories identified in the included studies, aggregated by framework/model to enhance practical usefulness. For each framework or model, the studies in which it was applied are listed, allowing readers to quickly identify how and where specific implementation approaches have been used in palliative care contexts.

| **Frameworks / Models / Theories** | **Study Name(s)** |
| --- | --- |
| Normalization Process Theory (NPT) | Lehmann et al. (2019); Bradshaw et al. (2021b) |
| Consolidated Framework for Implementation Research (CFIR) | Pinto et al. (2018); Lind (2018); Lehmann et al. (2019 – suggested) |
| Knowledge to Action (KTA) / Integrated Knowledge Translation (iKT) | Sawatzky et al. (2018); Schick-Makaroff et al. (2020); Howell et al. (2020) |
| Plan–Do–Study–Act (PDSA) / Model for Improvement | Spaner et al. (2017); Patel et al. (2022); Lee et al. (2016); Muir et al. (2018); Pezold et al. (2019); Rauenzahn et al. (2017) |
| Medical Research Council (MRC) Framework for Complex Interventions | Kane et al. (2017) |
| Promoting Action on Research Implementation in Health Services (PARIHS) | Diffin et al. (2018); Lind (2018) |
| Diffusion of Innovation Theory | Diffin et al. (2018) |
| Nonadoption, Abandonment, Scale-up, Spread and Sustainability (NASSS) | Hall et al. (2020) |
| Donabedian Model (Structure–Process–Outcome) | Potts et al. (2018) |
| Action Research | Hughes et al. (2004) |
| Continuous Quality Improvement / FOCUS–PDCA | Bookbinder et al. (1996) |
| Chronic Care and Related Service Models | Kane et al. (2018); van den Hurk et al. (2022) |
| Champion-Based Models (Nurse / Radiation Therapist) | Smith et al. (2017); Wu et al. (2022) |
| Micro–Meso–Macro Analytical Framework | Krawczyk et al. (2019b) |
| Specialty-Specific Care Models | Kilonzo et al. (2015); Voorend et al. (2021) |
