# Supplementary material for: Implementing patient-centred outcome measures in palliative care clinical practice. An updated systematic review of facilitators and barriers
Source: BMC Palliat Care. 2026 Feb 12;25:66. doi: 10.1186/s12904-026-01997-2 (PMC12997956; doi:10.1186/s12904-026-01997-2)
Supplement: Supplementary file 9 — Supplementary Material 9. [file 12904_2026_1997_MOESM9_ESM.docx]

# Appendix 9. Practical Overview for Implementing Patient-Centred Outcome Measures (PCOMs) in Palliative Care

This appendix consolidates evidence from Appendices 3–6 into a single, practice-oriented resource intended to support clinical teams planning to implement patient-centred outcome measures (PCOMs) in palliative care. Rather than providing exhaustive detail, it offers a structured overview of commonly used PCOMs, key facilitators and barriers to implementation, and cross-cutting lessons learned from empirical studies. The aim is to support informed, context-sensitive implementation that balances standardisation with person-centred care.

Understanding which PCOMs are most commonly used in palliative care can help teams make informed and pragmatic choices when selecting measures for routine practice.

| **Summary Box 1. Commonly Used PCOMs in Palliative Care**  • Most frequently used: ESAS / ESAS-r; POS; IPOS and adaptations  • Multidimensional, brief, and validated across settings  • Support clinical communication and shared decision-making  • Practice implication: prioritise a small number of fit-for-purpose PCOMs |
| --- |

Implementation success is strongly influenced by organisational, team, and process-related factors that enable PCOMs to be integrated into everyday clinical practice.

| **Summary Box 2. Key Facilitators of PCOM Implementation**  • Early planning and organisational readiness  • Visible leadership support and implementation champions  • Ongoing training and staff engagement  • Integration into workflows and electronic health records  • Flexible, patient-centred administration |
| --- |

Despite their recognised value, PCOMs are often difficult to implement. Awareness of common barriers can help teams anticipate challenges and plan mitigation strategies.

| **Summary Box 3. Key Barriers to PCOM Implementation**  • High workload, time pressures, and limited training  • Patient illness severity and fluctuating capacity  • Resource and technology limitations  • Measure complexity and interpretation challenges  • Organisational and system-level constraints |
| --- |

Across diverse settings, implementation studies provide valuable insights into what supports or hinders sustained PCOM use over time.

| **Summary Box 4. Lessons Learned from Implementation Studies**  • Implementation is iterative and long-term  • Contextual fit with teams and care models is critical  • Regular use increases perceived value  • Training must support interpretation and action  • Balance standardisation with person-centred flexibility  • Digital PCOMs enable integration but introduce equity and usability challenges |
| --- |

Drawing together the evidence, several practical messages emerge for teams seeking to embed PCOMs into routine palliative care.

| **Summary Box 5. Take-Home Messages for Clinical Teams**  • Select validated PCOMs aligned with clinical goals  • Invest in leadership, champions, and sustained training  • Embed PCOMs into everyday workflows  • Plan for ongoing evaluation and adaptation  • Ensure PCOM data are used meaningfully in patient care |
| --- |
